# Inflammatory Mechanisms in Acute Coronary Syndromes: From Pathophysiology to Therapeutic Targets

**DOI:** 10.3390/cells15010072

**Published:** 2025-12-31

**Authors:** Daniel Miron Brie, Cristian Mornoș, Ovidiu Adam, Alexandru Tîrziu, Roxana Popescu, Alina Diduța Brie

**Affiliations:** 1 Cardiovascular Disease Institute Timisoara, Gheorghe Adam Street, No. 13A, 300310 Timisoara, Romaniamornos.cristian@umft.ro (C.M.); 2 Research Center of the Institute of Cardiovascular Diseases, Gheorghe Adam Street, No. 13A, 300310 Timisoara, Romania; 3 Department of Cardiology, “Victor Babes” University of Medicine and Pharmacy, Eftimie Murgu Square, No. 2, 300041 Timisoara, Romania; 4 Department of Pediatric Surgery and Orthopedics, “Victor Babes” University of Medicine and Pharmacy, Eftimie Murgu Square, No. 2, 300041 Timisoara, Romania; ovidiu.adam@umft.ro; 5 Department of Functional Sciences, “Victor Babes” University of Medicine and Pharmacy, Tudor Vladimirescu Street, No. 14, 300174 Timisoara, Romania; 6 ANAPATMOL Research Center, “Victor Babes” University of Medicine and Pharmacy, Tudor Vladimirescu Street, No. 14, 300174 Timisoara, Romania; popescu.roxana@umft.ro; 7 Department of Cell and Molecular Biology, Faculty of Medicine, “Victor Babes” University of Medicine and Pharmacy, Eftimie Murgu Square, No. 2, 300041 Timisoara, Romania; alina.brie@umft.ro; 8 “Louis Țurcanu” Emergency Children Hospital, Doctor Iosif Nemoianu Street, No. 2, 300011 Timisoara, Romania

**Keywords:** acute coronary syndrome, inflammation, neutrophil extracellular traps, T lymphocytes, anti-inflammatory therapy, colchicine, interleukin-1, cardiovascular immunology

## Abstract

Inflammation plays a pivotal role in the pathogenesis of acute coronary syndromes (ACS), contributing to plaque instability, thrombosis, and myocardial injury. This review aims to comprehensively examine the inflammatory mechanisms underlying ACS and evaluate current and emerging anti-inflammatory therapeutic strategies. We conducted a comprehensive literature review examining the role of inflammatory pathways in ACS pathophysiology, including innate and adaptive immune responses, key inflammatory mediators, and cellular mechanisms. We analyzed current evidence for anti-inflammatory therapies and their clinical outcomes in ACS management. Inflammatory processes in ACS involve complex interactions between innate immune cells (neutrophils, macrophages, monocytes) and adaptive immune cells (T lymphocytes, B cells). Key mechanisms include neutrophil extracellular trap (NET) formation, macrophage polarization, T cell subset imbalances (Th1/Th17 predominance with regulatory T cell dysfunction), and complement activation. Inflammatory biomarkers such as C-reactive protein, interleukin-6, and NET-specific markers demonstrate prognostic value. Anti-inflammatory therapies including colchicine, canakinumab (IL-1β inhibition), and methotrexate have shown cardiovascular benefits in clinical trials. Emerging targets include NET inhibition, T cell modulation, and precision inflammatory profiling approaches. Inflammation represents a critical therapeutic target in ACS beyond traditional risk factor modification. While colchicine and IL-1β inhibition have demonstrated clinical efficacy, future strategies should focus on precision medicine approaches targeting specific inflammatory pathways based on individual patient profiles. Integration of anti-inflammatory therapy with lipid management and antithrombotic strategies offers promise for improving ACS outcomes through comprehensive targeting of the multifactorial pathophysiology underlying coronary artery disease.

## 1. Introduction

Inflammation plays a central role in the pathogenesis of acute coronary syndromes (ACS) by contributing to the initiation, progression, and complications of atherosclerotic plaques [1]. Local inflammatory cells in the coronary arteries release cytokines that activate the endothelium, altering its physiological antiaggregant and anticoagulant properties. This activation promotes plaque instability by reducing extracellular matrix synthesis and increasing its degradation, thereby favoring plaque rupture—a key event in the onset of ACS. Inflammatory cytokines also increase smooth muscle cell reactivity and contribute to thrombus formation following plaque disruption [2]. Both innate and adaptive immune responses regulate the progression of atherosclerosis and plaque stability. Immune cells, such as macrophages and T lymphocytes, secrete inflammatory mediators that enhance the lipid core while reducing the fibrous cap, thereby making plaques more prone to rupture. The 2023 European Society of Cardiology (ESC) guidelines for the management of acute coronary syndrome (ACS) acknowledge the role of inflammation in ACS pathophysiology but do not currently provide specific recommendations for targeting inflammation as a routine therapeutic strategy in ACS management [1].

A recent article discusses the inflammatory response in patients with acute myocardial infarction, drawing inspiration from the title of a famous Western movie, “The Good, the Bad and the Ugly” It is based on the idea that after an acute myocardial infarction, the initial inflammatory response plays a beneficial, defensive role, known as “The Good.” This response can be exaggerated in the acute phase, “The Ugly”. Additionally, a chronic inflammatory response may persist long after the acute event, thereby realizing the residual inflammatory risk, “The Bad” [3].

The paper demonstrates that inflammation is both beneficial and detrimental after an ACS. A certain amount of inflammation helps the heart heal by removing dead cells and supporting repair processes. On the other hand, too much inflammation can lead to additional damage and complications, such as worsening heart function or triggering additional ACS. Targeted treatments that reduce the harmful inflammation (often referred to as “The Bad” and “The Ugly”) while leaving the protective, healing inflammation (“The Good”) intact are necessary to aid in recovery after an acute coronary syndrome while preventing the onset of another one. This balance could improve recovery and reduce the risk of complications. The repair process following AMI is likely to involve M2-type macrophages, natural killer (NK) cells, regulatory T lymphocytes (Treg), and cytokines, specifically interleukin-10 (IL-10) and interleukin-2 (IL-2). The acute phase of the inflammatory response is characterized by the activation of polymorphonuclear leukocytes (PMNs), M1 macrophages, the formation of neutrophil extracellular traps (NETs), and the release of cytokines, including interleukin-1α (IL-1α), interleukin-1β (IL-1β), and interleukin-6 (IL-6). After this acute phase, a chronic inflammatory process may develop, resulting in a residual inflammatory risk (RIR). This process involves M1-polarized macrophages, foam cells, and PMNs, as well as the activation of the NLRP3 inflammasome.

So, in the acute phase after a myocardial infarction, anti-inflammatory treatment should protect or stimulate the inflammatory repair processes and limit the acute exaggerated inflammatory response.

This review focuses on the current state of research regarding anti-inflammatory medication in patients with acute myocardial infarction. We want to answer questions such as: What drugs are currently available? What drugs will be available? Will they change treatment guidelines? When is the best time after an acute myocardial infarction to initiate anti-inflammatory treatment? Is anti-inflammatory treatment applicable to all patients with acute coronary syndromes?

## 2. Inflammatory Mechanisms Involved in Atherosclerotic Plaque Destabilization

Atheromatous plaques that are prone to rapid progression, rupture, or erosion leading to ACS are referred to as vulnerable plaques, as they have specific, well-defined characteristics [4,5]. The term “vulnerable plaque” was first used by Muller et al., who described plaque prone to rupture leading to ACS, histopathologically characterized (based on autopsy studies) by a lipid-rich core, a thin cap fibroatheroma, and an inflammatory infiltrate with macrophages [6]. Although atherosclerotic plaque rupture is the most incriminated mechanism in the pathophysiology of ACS, plaque erosion or the presence of calcified nodules have been described as possible mechanisms [7]. Nowadays, it is recommended to use the term vulnerable plaque to define those atherosclerotic plaques that can cause complications, being used in the designation of all plaques at risk of rupture and thrombosis [8]. An expert consensus proposes several major criteria (active inflammation, a thin cap with a large lipid core, endothelial denudation with superficial platelet aggregation, fissured plaque, and stenosis > 90%) and minor criteria (superficial calcified nodules, glistening yellow aspect on angioscopy, intraplaque hemorrhage, endothelial dysfunction, and outward (positive) remodeling) for diagnosing vulnerable plaque. The vast majority of plaques that cause an ACS are without calcifications, no significant stenosis, or similar to type IV atherosclerotic lesions described in the American Heart Association classification (a well-defined vascular intimal region occupied by extracellular lipids without a significant amount of fibrous tissue and without surface erosion, rupture or thrombus formation) [9].

Inflammation plays a central role in the pathogenesis of ACS, with various inflammatory markers and pathways contributing to disease progression and outcomes.

### 2.1. NOD-like Receptor Protein Domain-Associated Protein 3 (NLRP3) Inflammasome

The NOD-like receptor protein domain-associated protein 3 (NLRP3) inflammasome plays a critical role in the development and progression of ACS by mediating inflammatory responses that contribute to plaque instability, myocardial injury, and adverse cardiac remodeling [10]. The NLRP3 inflammasome consists of the NLRP3 protein functioning as a sensor for pattern-recognition repeats, danger and pathogen-associated molecular patterns (DAMPs, PAMPs), an adaptor domain (ASC—a caspase-recruiting domain, CARD), and an effector protein (caspase 1). The sensor domain includes an N-terminal pyrin domain, a central NACHT domain vital for ATP-mediated inflammasome assembly and a leucine-rich repeat domain (LRR) [11].

Upon activation by danger signals such as cholesterol crystals, potassium efflux, or ischemic injury, the NLRP3 oligomerizes with the NACHT domains, which further rectruits ASC, which attach to the ASC speck. The activated inflammasome activates caspase 1, which cleaves the pre-IL-1β and pre-IL-18 into their active forms. As a result, IL-1β and IL-18 are released, promoting local vascular inflammation by upregulating the ICAM-1 and VCAM-1 molecules on endothelial cells, promoting immune cell adhesion and infiltration, further contributing to the development and destabilization of atherosclerotic plaques. Additionally, caspase-1 mediates activation of Gasdermin D, which upon oligomerization, forms a plasmallemal pore, triggering pyroptosis. NLRP3 activation can precipitate ACS events [12] by enhancing the lipid necrotic core and driving IL-1/IL-18-mediated vascular inflammation (pyroptosis) and may constitute a therapeutic target in patients with ACS [13,14].

The inflammasome-driven cytokines exacerbate inflammation within plaques, weakening the fibrous cap through matrix metalloproteinase activation and increasing the likelihood of plaque rupture or erosion, the primary triggers of ACS. Beyond plaque effects, NLRP3 activation in cardiac cells (cardiomyocytes and fibroblasts) during ischemia promotes inflammatory cell death (pyroptosis) and fibrosis. This contributes to adverse ventricular remodeling and impaired cardiac function after myocardial infarction, worsening prognosis [15]. Molecular mechanisms, including microRNAs and protein modifications (such as SUMOylation, phosphorylation and ubiquitylation) tightly regulate NLRP3 activation [16]. Pharmacological inhibition of NLRP3 or its downstream cytokines (e.g., IL-1β) has shown promise in reducing inflammation, limiting cardiac damage, and improving outcomes in preclinical and early clinical studies [13,17].

A recent study highlights the role of the inflammasome signaling pathway in myocardial damage during STEMI. By identifying the genetic expression of inflammasome-related markers like TLR4, NLRP3, and IL-6R in coronary thrombi, the research suggests that these markers are actively involved in the inflammatory response associated with myocardial injury. The findings support targeting the inflammasome signaling pathway as a potential treatment strategy for myocardial infarction. By focusing on this pathway, medical interventions could potentially reduce inflammation and limit heart damage, improving patient outcomes, but the timing of anti-inflammatory treatment is crucial [18].

### 2.2. Interleukin-1 β (IL-1β)

IL-1β is a pro-inflammatory cytokine that is activated through the NLRP3 inflammasome pathway. IL-1β is highly expressed in atherosclerotic plaques, particularly in complicated and unstable plaques, prone to rupture. IL-1β promotes inflammation by inducing the expression of adhesion molecules (ICAM-1, VCAM-1), chemokines, and matrix metalloproteinases (MMP-9), which degrade the fibrous cap and increase plaque vulnerability [19]. Studies have shown that IL-1β levels are elevated in patients with ACS and correlate with myocardial damage and adverse outcomes. IL-1β stimulates other proatherogenic cytokines such as TNF-α and LIGHT, amplifying vascular inflammation and contributing to plaque progression and rupture [20]. IL-1β exerts direct harmful effects on heart muscle cells by inducing apoptosis (programmed cell death) and hypertrophy, depressing cardiac contractility through nitric oxide-dependent and independent pathways [21]. IL-1β promotes leukocyte trafficking to the site of myocardial injury by enhancing endothelial adhesion molecule expression and chemokine production, which sustains the inflammatory response after myocardial infarction. This inflammatory cascade contributes to infarct healing but can also lead to excessive tissue damage and adverse cardiac remodeling, if uncontrolled [22].

### 2.3. Interleukin-6 (IL-6)

IL-6 is elevated in patients with ACS, especially in those with unstable angina and acute myocardial infarction, reflecting increased inflammatory activity within atherosclerotic plaques [23]. It is expressed locally in the shoulder regions of plaques, where it enhances recruitment of monocytes and T cells, promoting inflammation and weakening the fibrous cap, thus increasing plaque vulnerability and risk of rupture [24].

IL-6 induces hepatic production of acute-phase reactants such as high-sensitivity C-reactive protein (hs-CRP), amplifying systemic inflammation and generating a pro-thrombotic state [25,26,27]. Higher plasma IL-6 levels are correlated with a greater severity of ACS. They are associated with an increased risk of major adverse cardiovascular events (MACE), including cardiovascular death, myocardial infarction, and hospitalization for heart failure [28]. IL-6 levels provide prognostic information that is independent of traditional risk factors and biomarkers, such as hs-CRP and troponin. Patients in the highest IL-6 quartile have significantly worse outcomes [29]. IL-6 activates the MAPK/ERK1/2 pathway, leading to overexpression of the MMP9, which contributes to extracellular matrix remodeling and fibrosis, processes involved in adverse ventricular remodeling and heart failure following myocardial infarction [27]. IL-6 also activates transcription factors such as NF-κB and PPAR-γ, which contribute to MMP-9 gene transcription in vascular cells and macrophages, promoting inflammatory responses and ECM degradation in atherosclerotic plaques [30].

### 2.4. Tumor Necrosis Factor α (TNF-α)

TNF-α, abundantly expressed in unstable atherosclerotic plaques, exacerbates local inflammation by activating endothelial cells and recruiting immune cells, thereby promoting plaque vulnerability and increasing the risk of rupture that precipitates acute coronary syndrome (ACS). TNF-α binds platelet TNFR1, activating the arachidonic acid cascade to increase thromboxane A2 production, which promotes platelet activation and aggregation, leading to thrombus formation that can occlude coronary arteries and cause myocardial ischemia [31,32]. Cardiac myocytes produce TNF-α during ischemia that has direct deleterious effects on heart muscle cells, including apoptosis and depressing contractile function [33]. TNF-α binds to TNF receptor 1 (TNFR1) on cardiomyocytes, triggering recruitment of adaptor proteins that activate caspase-8. Activated caspase-8 initiates the apoptotic cascade directly cleaves Bid into truncated Bid (t-Bid), which translocates to mitochondria, promoting the formation of the mitochondrial outer membrane permeabilization pore and release of pro-apoptotic factors such as cytochrome c, Smac/Diablo, and Omi/HtrA2 into the cytosol. Cytochrome c activates caspase-9 which, together with caspase-8, activates caspase-3, the executioner caspase responsible for apoptosis [34,35]. This pathway contributes to left ventricular dysfunction and adverse remodeling after myocardial infarction. Elevated circulating TNF-α levels in ACS patients, including several months after myocardial infarction, are associated with an increased risk of recurrent coronary events, heart failure, and mortality. Persistently high TNF-α levels indicate ongoing inflammatory instability and a worse prognosis [36,37].

### 2.5. Interleukin-18 (IL-18)

Patients with ACS show significantly elevated circulating IL-18 levels compared to healthy controls. IL-18 concentrations rise rapidly after the acute event and could remain elevated for months, indicating sustained inflammation [38].

IL-18, like IL-1β, is released during the NLRP3-mediated pyroptosis and amplifies vascular inflammation [39]. It promotes the recruitment and activation of immune cells within atherosclerotic plaques, contributing to plaque destabilization and increasing the risk of rupture and thrombosis, which trigger ACS.

IL-18 binds to IL-18Rα, activating NF-κB signaling pathways in vascular cells and macrophages, upregulating the MMP-9 (leading to extracellular matrix remodelling) and the CD36, a multiscavenger receptor that binds and internalizes oxidized LDL-cholesterol lipoprotein particles, favoring foam cell formation [40]. IL-18 also upregulates the production of interferon-γ, further activating macrophages and T/NK cells. Interferon-γ down-regulates collagen production by the vascular smooth muscle cells, leading to plaque fibrous cap thinning, making the plaque more unstable and prone to rupture [41].

Elevated IL-18 levels are associated with a higher risk of adverse clinical events after ACS, including recurrent unstable angina, myocardial infarction, heart failure exacerbation, stroke, and cardiovascular death. IL-18 levels above the median significantly predict worse outcomes, independent of many clinical factors [42]. However, its prognostic value is somewhat attenuated when adjusted for other inflammatory biomarkers, suggesting shared inflammatory pathways. Higher IL-18 levels correlate with male sex, obesity, diabetes, decreased renal function, and other markers of inflammation, reflecting its role in systemic inflammatory burden in ACS patients [39,43].

### 2.6. Chemokines in Acute Coronary Syndrome

Chemokines are chemotactic molecules that play a role in recruiting immune cells to sites of inflammation. Chemokines influence the migration of leukocytes, including neutrophils, monocytes, and T cells, into atherosclerotic plaques. This influx of inflammatory cells is a hallmark of plaque destabilization and rupture, which are critical events leading to ACS [44,45]. Beyond recruiting immune cells, chemokines directly contribute to the weakening of the fibrous cap of atherosclerotic plaques by inducing the release of matrix-degrading enzymes from macrophages, which degrade the extracellular matrix and promote apoptosis of endothelial cells and smooth muscle cells, further compromising plaque stability [46]. Chemokines can enhance the pro-thrombotic environment within plaques by inducing tissue factor expression in vascular smooth muscle and endothelial cells and promoting platelet activation and aggregation, which are essential steps in the formation of occlusive thrombi following plaque rupture. Elevated levels of various chemokines, including CCL2, CCL3, CCL5, CCL18, CCL19, and CCL21, have been observed in patients with acute coronary syndrome (ACS) and are associated with worse outcomes. These chemokines not only reflect ongoing inflammation but also actively participate in amplifying the inflammatory response within the coronary arteries [47].

CCL2 (prognostic biomarkers and possible therapeutic target) determines monocyte recruitment, plaque destabilization, and predicts major cardiovascular events. CCL2 expression and signaling activate the NF-κB and p42/44 MAPK (ERK1/2) pathways in endothelial cells and monocytes, promoting transcription of proinflammatory genes and adhesion molecules (VCAM-1, ICAM-1) that enhance leukocyte recruitment and retention [48]. In addition to CCR2, CCL2 binds to atypical receptors like ACKR1, which do not induce cell migration but modulate chemokine gradients and inflammatory responses within plaques, influencing local CCL2 activity and plaque microenvironment [49]. Furthermore, CCL2 interacts with other chemokines (CCL4) and receptors (CCR5), activating transcription factors c-Jun and c-Fos, which may regulate CCL2 expression and amplify inflammatory signaling [50].

CCL3, CCL5, and CCL18 determine leukocyte recruitment, and are independently associated with a higher short-term mortality risk [51,52].

Plasma levels of CCL3 are significantly increased in patients with acute myocardial infarction (AMI) and unstable angina, rising rapidly after ischemic events and remaining elevated for months, reflecting ongoing inflammation [53]. CCL3 is produced mainly by leukocytes (macrophages and neutrophils) during inflammation and promotes chemotaxis of neutrophils, T cells, and macrophages to atherosclerotic plaques and ischemic myocardium. This recruitment exacerbates local inflammation and tissue injury. The association between CCL13, vascular inflammation, neutrophil infiltration and plaque progression was proven using CCL13/ApoE-deficient mice expressing lower levels of circulating neutrophils, lower neutrophil infiltration in atherosclerotic plaques and a lower responsivity to CXCL1 when fed with Western-type diet [54].

CXCL1 is highly upregulated in response to ischemic cardiac injury and angiotensin II stimulation. It binds to CXCR2 on monocytes and neutrophils, promoting their chemotaxis into the heart tissue. CXCL1–CXCR2 engagement activates NF-κB and p42/44 MAPK (ERK1/2) pathways in immune and cardiac cells, leading to transcription of proinflammatory cytokines, adhesion molecules (VCAM-1, ICAM-1), and matrix metalloproteinases (MMPs) [48]. CXCL1–CXCR2 signaling contributes to activation of NADPH oxidase (NOX2), which increases ROS production, promoting oxidative stress and further inflammation in cardiac tissues [55].

At the cardiac level, CXCL1 promotes differentiation of cardiac fibroblasts into myofibroblasts, increasing collagen deposition and fibrosis, which impair cardiac function and contribute to heart failure progression. Additionally, by interacting with the Smad2/3 and TGF-β pathways, it leads to pathological atrial remodelling, increasing the susceptibility to atrial fibrillation [55,56]. However, at the vascular level, it exerts a protective role via the same pathway by making the plaque less susceptible to rupture.

Although CXCL5 was more intensively studied in the process of tumor progression, recent efforts have been made to identify its roles in atherosclerotic disease. Similar to CXCL1, CXCL5 binds to CXCR2 on neutrophils and monocytes, promoting their chemotaxis to sites of injury and inflammation [57]. This recruitment amplifies local inflammation and tissue damage. CXCL5 activates intracellular signaling cascades including NF-κB and AKT, which promote transcription of proinflammatory genes, survival signals, and angiogenic factors [58]. In endothelial cells, CXCL5 induces NF-κB-mediated expression of vascular endothelial growth factor A (VEGF-A) via the FOXD1 transcription factor, enhancing angiogenesis and vascular repair processes post-injury. CXCL5 stimulates the MAPK/ERK pathway, which regulates cell proliferation, migration, and survival [59]. This pathway contributes to endothelial cell activation and smooth muscle cell migration. CXCL5 activates epithelial-to-mesenchymal transition (EMT)-related pathways (ERK/Elk-1/Snail and PI3K/AKT/GSK-3β/Snail-Twist), which may have parallels in vascular remodeling and fibrosis after myocardial injury and influencing the development of collateral circulation after an acute coronary syndrome [60]. Supporting evidence for its protective role in coronary artery disease was provided by Ravi et al., who identified a negative correlation between circulating CXCL5 levels and the severity of coronary artery disease, independent of statin use, gender nor age [61].

## 3. Immune Cells in Acute Coronary Syndrome

### 3.1. Monocytes and Macrophages

Monocytes are recruited to the arterial wall, where they differentiate into macrophages and phagocytose lipids, becoming foam cells. Foam cells will secrete proinflammatory cytokines, such as IL-1β and TNF-α, which contribute to plaque instability and rupture. Macrophages can polarize into two main phenotypes: M1 (proinflammatory) macrophages, which promote inflammation and plaque vulnerability, and M2 (anti-inflammatory) macrophages, which are more prevalent in advanced plaques and may have protective roles [62,63,64].

Macrophage infiltration is a crucial and early step in the formation of atherosclerotic plaques. Monocytes migrate into the intima, differentiate into macrophages, and take up oxidized LDL via scavenger receptors (e.g., SR-A, CD36), transforming into foam cells. These foam cells and macrophages produce inflammatory cytokines, such as IL-6 and TNF-α, as well as matrix metalloproteinases, particularly MMP-1 and MMP-9, which degrade extracellular matrix proteins. This degradation weakens the fibrous cap of plaques, making them more prone to rupture and thrombus formation, which can precipitate acute coronary syndrome (ACS) events [65]. Monocytes/macrophages secrete proinflammatory cytokines that activate neighboring vascular smooth muscle cells, promoting the formation of the extracellular matrix, fibrosis, and further destabilization of the plaques.

The inflammatory milieu created by these cells amplifies local immune responses, attracting more immune cells and sustaining chronic inflammation within the plaque [66]. Monocytes, with their various subpopulations, play a central role in both inflammation and repair mechanisms within the cardiovascular system. Specific subsets of monocytes may promote inflammation, contributing to tissue injury, while others are linked to repair and healing processes after myocardial damage. After MI, monocytes are rapidly recruited to the ischemic heart tissue, where they differentiate into inflammatory macrophages. These macrophages clear dead cells and extracellular debris through phagocytosis, and they secrete proteases and reactive oxygen species, which are essential for tissue remodeling but can also exacerbate injury if produced in excessive amounts [67,68].

The number of monocytes/macrophages increases significantly in the first hours after an MI, and their level remains elevated for about two weeks after an MI. Initially, the infiltrate has a pro-inflammatory role that gradually transitions into a reparative one, contributing to scar formation [64,69].

### 3.2. Neutrophils

Polymorphonuclear neutrophils (PMNs) play a significant and multifaceted role in ACS, primarily through their involvement in inflammation, plaque destabilization, and thrombosis. These cells are among the first responders to vascular injury, releasing proteolytic enzymes and reactive oxygen species that exacerbate endothelial damage and promote thrombosis. Neutrophils are actively recruited into atherosclerotic plaques, particularly in unstable lesions underlying ACS, such as AMI. Endogenous activation of Toll-like receptor 2 (TLR2) on endothelial cells promotes neutrophil recruitment and accumulation at sites of early plaque erosion, as demonstrated in murine models. They are found in significantly higher numbers in ruptured and eroded plaques compared to stable plaques, suggesting their direct involvement in plaque destabilization [70]. Activated neutrophils release enzymes such as neutrophil elastase, myeloperoxidase (MPO), and matrix metalloproteinases (MMPs). These enzymes degrade extracellular matrix components and basement membranes, contributing to endothelial damage and weakening of the fibrous cap, which can precipitate plaque rupture and thrombus formation. Activated neutrophils can release Neutrophil Extracellular Traps (NETs), web-like structures composed of decondensed chromatin (DNA and histones) decorated with neutrophil granule proteins, such as myeloperoxidase and neutrophil elastase. These NETs provide a matrix that traps platelets, red blood cells, and coagulation factors, facilitating clot formation in coronary arteries and inducing endothelial cell injury and dysfunction, which in turn destabilizes the plaques. NETs stimulate sterile inflammation by activating immune cells and releasing inflammatory mediators, exacerbating vascular injury [71]. NETs form a scaffold within coronary thrombi and correlate positively with infarct size, indicating their role in worsening myocardial injury. DNase, an enzyme that degrades NETs, is associated with reduced infarct size, suggesting a protective mechanism against NET-mediated damage [72].

The ROS production during ischemia activates peptidyl-arginine-deiminase-4 (PAD4), an enzyme that facilitates citrullination of arginine residues in histones (particularly H3 histone), leading to chromatin decondensation. Chromatin unfolding occurs due to the disruption of the ionic interactions between the positively charged arginine residues and the negative charges from the DNA phosphate groups. PAD4 activation also favors translocation of neutrophil elastase and myeloperoxidase into the nucleus that attach to chromatin strands forming NETs. Finally, the nuclear and plasma membranes are lysed, expelling NETs into the extracellular compartment [73].

Increased plasma levels of MPO are associated with an increased risk of coronary artery disease [74], and MPO-DNA complexes are positively correlated with severe adverse cardiovascular events [75].

NET formation is implicated in multiple steps of ACS pathophysiology, from plaque destabilization and arterial thrombosis to reperfusion injury and impaired microvascular flow [76,77]. NETs were identified in human atherothrombotic plaques and retrieved coronary thrombi, being linked to a higher risk of cardiac-related death and stent thrombosis. NETs are also associated with microvascular obstruction and no-reflow after reperfusion, worsening infarct expansion [78,79].

Coronary blood flow restoration during fibrinolysis or PCI increases vascular inflammation significantly, activating the neutrophils which release NETs through the suicidal pathway, augmenting the inflammatory response in a positive feedback fashion [80]. Activated neutrophils express a higher NADPH oxidase activity, which generates reactive oxygen species. ROS are crucial for the formation of NETs by breaking down nuclear and cellular membrane to allow NET release. However, the local oxidative stress also affects myocardial tissues, augmenting cardiac muscle damage and release of pro-inflammatory molecules [81,82]. Excessively accumulated NETs in the ischemic/necrotic myocardium favor the formation of microthrombi by interacting with red blood cells and platelets, contributing to the ‘no-reflow’ phenomenon [83].

Neutrophils also contribute to the inflammatory milieu by releasing cytokines (IL-1β and IL-18) and interacting with other immune cells, such as macrophages, thereby amplifying local inflammation within the plaque [82].

Elevated circulating neutrophil counts and neutrophil/lymphocyte ratios are associated with unstable coronary artery disease and predict worse cardiovascular outcomes in ACS patients. Neutrophil levels on admission correlate with mortality and major adverse cardiac events, making them useful prognostic markers [84].

### 3.3. T Lymphocytes

T-cells, especially CD4+ helper T-cells, are more frequently activated in ACS compared to stable angina. Single-cell RNA sequencing has revealed distinct clusters of CD4+ T cells, including effector, naive, cytotoxic, central memory, and regulatory T cells [85]. In ACS, there is a shift toward aggressive effector phenotypes with a high frequency of T helper cell 1 (Th1) and a reduction in regulatory T-cells (Tregs), which normally suppress excessive immune responses [86]. They have a lower activation threshold due to amplified T-cell receptor (TCR) signaling, including increased accumulation of signaling molecules like CD3 complexes, ZAP70, and defective deactivation of LCK kinase [87]. CD4+ T-cells in ACS patients show enhanced activation, lower thresholds for stimulation, and increased cytotoxicity, even capable of killing endothelial and vascular smooth muscle cells [86].

#### 3.3.1. Th1 Cells

Th1 cells, characterized by T-bet expression and IFN-γ production, promote macrophage activation, endothelial dysfunction, and pro-inflammatory plaque phenotypes [88]. Clinical studies demonstrate Th1 enrichment in acute myocardial infarction (AMI) patients, with elevated Th1 frequencies correlating with systemic inflammation markers and plaque vulnerability indices [89]. The IFN-γ produced by Th1 cells mediates pro-atherogenic endothelial and macrophage phenotypes, contributing to plaque destabilization [90].

#### 3.3.2. Th17 Cells

Th17 cells, expressing RORγ and producing IL-17A, facilitate neutrophil recruitment and matrix degradation through metalloproteinase induction [86]. A higher Th17 number is linked to plaque vulnerability and functional decline after ACS [91]. The Th17 pathway can activate matrix-metalloproteinases with atheroma cap thinning, potentially contributing to plaque rupture events.

#### 3.3.3. Regulatory T Cells (Tregs)

Tregs, characterized by FOXP3 expression and IL-10/TGF-β production, suppress effector T cell responses and promote tissue repair. In ACS patients, reduced or dysfunctional Tregs correlate with worse clinical outcomes and impaired post-infarction remodeling. Tregs support scar formation and healing by inducing macrophage phenotypic change towards the M2 phenotype and limiting excessive inflammatory responses via IL-10 and TGF-β release [92].

#### 3.3.4. CD8+ T Cells

CD8+ T cells exert significant cytotoxic functions in both infarcted myocardium and atherosclerotic plaques. These cells kill target cells via perforin/granzyme and Fas-FasL pathways. Granzyme B-dependent CD8+ activity is linked to adverse remodeling after ischemic injury [93]. Expanded CD4+ CD28-/CD28null and activated CD8+ subsets with effector/senescent features accumulate in the coronary circulation during STEMI and are associated with increased local inflammation [94].

The activation of CD8+ T cells requires TCR recognition of peptide-MHC complexes plus co-stimulation (CD28/B7, CD40/CD40L), and is modulated by immune checkpoints such as CTLA-4 and PD-1 that restrain pathogenic responses [95]. By inhibiting the co-stimulatory axes (CD40 inhibitors, CD28 inhibitors) or augmenting the inhibitory checkpoints (CTLA-4, PD-1 agonists), T cell-mediated vascular inflammation may be mitigated.

**Figure 1:**
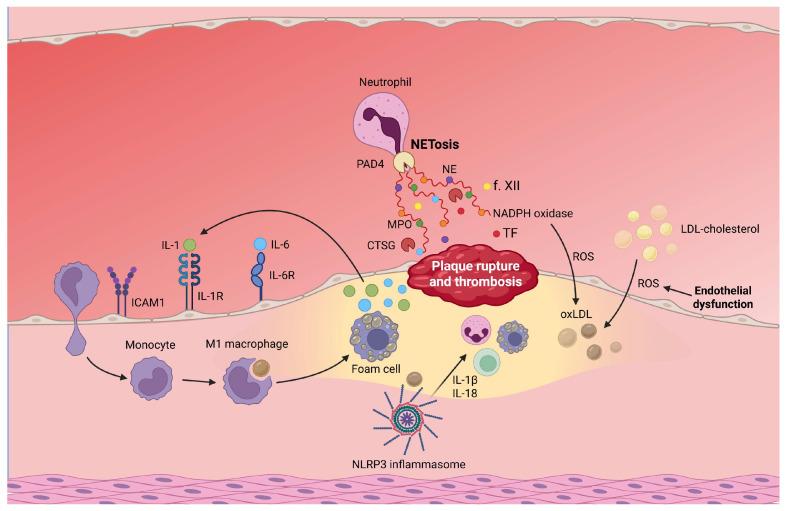
Schematic representation of atherosclerotic plaque rupture and thrombosis. Created in BioRender. Tirziu, A. (2025), https://BioRender.com/mmejda6 [Accessed on 9 November 2025].

The mechanism of atherosclerosis and plaque rupture is summarized in Figure 1.

## 4. Inflammatory Markers in Acute Coronary Syndrome

### 4.1. C-Reactive Protein (CRP)

CRP is an acute-phase protein produced mainly by hepatocytes in response to inflammatory cytokines, including interleukin-1 and interleukin-6 [96]. Its levels rise significantly during ACS, reflecting systemic and vascular inflammation linked to plaque instability and myocardial injury. Elevated CRP levels on admission in ACS patients are strongly associated with worse short- and long-term cardiovascular outcomes, including higher risk of death, myocardial infarction, heart failure, and MACE. In healthy individuals, CRP levels typically range from about 1.2 to 2.0 mg/L [97]. CRP levels start increasing around 6 h after symptom onset in ACS, typically reach peak values between 36 and 50 h (approximately 1.5 to 2 days) after the event [98].

Patients with CRP levels above 3 mg/L have significantly lower survival rates and a higher incidence of complications [99,100]. CRP levels ≥ 10 mg/L are commonly used as a cutoff to indicate significant inflammation and a higher risk of adverse outcomes. Elevated CRP above 10 mg/L within 24 to 48 h after ACS onset is associated with worse prognosis and increased long-term mortality [98,100].

CRP levels tend to increase following myocardial injury, with higher levels observed in STEMI and NSTEMI compared to unstable angina. The magnitude of CRP elevation correlates with the extent of myocardial necrosis and inflammatory response [98]. Measuring CRP, especially high-sensitivity CRP (hs-CRP), helps identify patients at higher risk who may benefit from more aggressive treatment and closer monitoring post-ACS. Combining CRP with other markers, such as troponin, enhances risk stratification. Studies have shown that patients who achieve lower CRP levels after treatment, especially with statins, have better outcomes. For example, in the PROVE IT-TIMI 22 trial, ACS patients who reduced their CRP levels below 2 mg/L after intensive statin therapy had significantly lower rates of death or recurrent MI compared to those with CRP levels above 2 mg/L, independent of cholesterol levels [101,102]. Persistently elevated CRP after ACS, even with modern therapies, indicates ongoing inflammation and predicts worse long-term prognosis, including higher mortality and heart failure risk [103]. CRP measurement can thus be used to monitor the inflammatory response and treatment efficacy, guiding more aggressive or tailored therapy in patients with persistently high CRP post-ACS.

### 4.2. Elevated White Blood Cell and Neutrophil Counts in ACS

Studies consistently show that elevated white blood cell (WBC) counts at admission in ACS patients are linked to higher risks of mortality, recurrent myocardial infarction, and MACE such as cardiac death and target lesion revascularization [104]. Elevated WBC count is an independent predictor of both short-term and long-term mortality in ACS, including STEMI and non-STEMI patients. Patients with higher WBC counts have a significantly increased risk of death during hospitalization and follow-up periods [105]. Higher WBC and neutrophil counts correlate with more severe coronary artery disease, including the presence of active thrombus, multi-vessel disease, and reduced LVEF. Elevated counts are also associated with complications such as congestive heart failure and cardiogenic shock [106].

Higher baseline WBC counts are associated with increased risk of ischemic events (cardiovascular death, MI, stroke) at 30 days and 1 year post-ACS [107].

The neutrophil-to-lymphocyte ratio (NLR) is the commonly used neutrophil ratio in ACS. It is calculated by dividing the absolute neutrophil count by the absolute lymphocyte count from a routine complete blood count. NLR is a simple, widely available, and cost-effective marker of systemic inflammation that has substantial predictive value for myocardial damage, cardiac dysfunction, rehospitalization, and mortality in ACS patients [108,109]. Elevated NLR reflects heightened inflammation (neutrophilia) combined with relative immunosuppression (lymphopenia), making it a robust biomarker for predicting short- and long-term adverse cardiovascular outcomes in ACS. An NLR cutoff of around 2.5 is commonly used for diagnosing ACS, with a reported sensitivity of about 63.6% and specificity of 80.2%. An NLR exceeding approximately 2.76 is associated with heightened myocardial damage and diminished cardiac function, as evidenced by reduced ejection fraction and fractional shortening. Elevated thresholds, such as NLR > 4 or >4.5, have been linked to adverse outcomes, including increased in-hospital mortality and major adverse cardiac events (MACE). Stratification of NLR into tertiles (e.g., <2.6, 2.6–4.5, >4.5) correlates with escalating risk and disease severity. Furthermore, NLR demonstrates a positive correlation with established clinical risk scores, such as GRACE and SYNTAX, which evaluate prognosis and coronary lesion complexity in acute coronary syndrome (ACS) [110].

Studies demonstrate that higher NLR values are strongly associated with elevated cardiac troponin levels, including high-sensitivity troponin I (hsTnI), which is a sensitive marker of myocardial necrosis. NLR can predict troponin positivity in follow-up, with a cutoff around 2.8 showing good sensitivity (79%) and specificity (73%) for troponin elevation [111]. The combined assessment of NLR with troponin and CRP enhances risk stratification in ACS. Patients with high NLR and CRP levels have significantly higher rates of mortality and major adverse cardiac events (MACCE) compared to those with low levels.

### 4.3. IL-6

IL-6 levels are elevated in patients with ACS, including acute myocardial infarction and unstable angina, but not in stable angina [27,112]. IL-6 is a powerful predictor of long-term cardiovascular mortality and adverse events after ACS, outperforming traditional markers such as hsCRP. Elevated IL-6 levels are independently associated with increased risk of MACE, cardiovascular death, heart failure, and recurrent myocardial infarction [29]. IL-6 measurement is increasingly used in clinical practice to assess inflammatory status and stratify risk in ACS patients, as it may help identify patients who would benefit from more aggressive or targeted anti-inflammatory therapies [28]. The normal value of IL-6 in healthy adults is generally considered to be less than 5 pg/mL, with some sources citing a range up to about 7 pg/mL depending on the laboratory and assay used. A meta-analysis performed on healthy adults found a pooled average IL-6 level around 5.2 pg/mL (95% CI: 4.6–5.7 pg/mL), with values ranging from 0 to about 43.5 pg/mL in some individuals without apparent disease [113]. Patients with unstable angina have IL-6 levels about three times higher than healthy controls. Higher IL-6 levels are strongly associated with more severe ACS, including STEMI, MACE, cardiovascular death, heart failure, and recurrent myocardial infarction [27]. Reported levels in ACS can vary widely but typically fall between 10 to 50 pg/mL, sometimes much higher depending on severity and timing. A study in patients with STEMI showed IL-6 levels as high as 30–100 pg/mL shortly after symptom onset [114]. Elevated IL-6 levels are associated with a larger infarct size, higher risk of MACE, and worse short- and long-term prognosis. IL-6 levels rise within hours after the onset of myocardial injury and usually peak at 6–24 h, then gradually decline over a few days [115].

### 4.4. TNF α

The determination of tumor necrosis factor-α (TNF-α) in the context of ACS is primarily associated with its role as both a marker and mediator of inflammation, which is pivotal in the pathogenesis and complications of ACS. Patients with ACS, including those with unstable angina and acute MI, exhibit significantly elevated levels of TNF-α compared to control groups. This elevation is correlated with the severity of the condition and the incidence of complications such as recurrent angina, hemodynamic instability, and electrical abnormalities [116].

TNF-α plays a critical role in the progression of ischemia by promoting the release of endothelial adhesion molecules, activating leukocytes, and inducing the secretion of thrombocyte-activating factors. These processes collectively exacerbate coronary artery disease and contribute to plaque instability. Elevated levels of TNF-α are recognized as an independent predictor of adverse coronary events and mortality, alongside other inflammatory and injury markers such as IL-6, CRP, and troponin-T.

In patients with ACS who also have rheumatoid arthritis (RA), TNF-α expression on atherosclerotic plaques is even stronger, suggesting a more intense and sustained pro-inflammatory response that may worsen outcomes. Monitoring TNF-α in such patients could guide preventive strategies [32].

Beyond its role as a biomarker, TNF-α signaling influences cardiac physiology and disease processes, mediated partly through NF-κB pathways, highlighting its dual role in inflammation and cardiac tissue response [117].

Additionally, TNF-α levels and genetic polymorphisms have been linked to suicidal ideation in ACS patients, especially in the first two weeks after ACS indicating that TNF-α may also be involved in neuropsychiatric complications post-ACS [118].

The normal value of TNF-α in acute coronary syndrome (ACS) patients is not universally fixed, but studies show that TNF-α levels are significantly elevated in ACS compared to healthy controls. For example, one study found a marked increase in TNF-α levels in ACS patients, with higher levels particularly in those with acute myocardial infarction (MI) compared to unstable angina, and these elevated levels correlated with greater complications [119].

While exact numeric reference ranges vary by assay and population, TNF-α levels in healthy individuals are generally low (often <10 pg/mL), whereas ACS patients often exhibit significantly higher levels, sometimes several-fold above normal baseline values [118].

### 4.5. Novel Biomarkers in ACS

No blood biomarkers are exclusively specific to coronary vascular inflammation. HsCRP and IL-6 lack specificity for coronary artery disease (CAD), as they reflect systemic inflammation influenced by infections, obesity, or other conditions, limiting their diagnostic accuracy for isolated coronary tree inflammation [120].

However, lipoprotein-associated phospholipase A2 (Lp-PLA2) and myeloperoxidase (MPO) are more relevant to coronary disease, as they target plaque-specific processes, such as the hydrolysis of oxidized phospholipids and neutrophil activation.

Lp-PLA2 localizes to atherosclerotic plaques, particularly in macrophages and necrotic cores, where it amplifies local vascular inflammation rather than reflecting systemic processes like hsCRP. Elevated levels correlate with high-risk plaque features on CT angiography, such as thin fibrous caps and positive remodeling, independent of traditional lipids [121]. Lp-PLA2 predicts coronary events (e.g., ACS) after adjusting for CRP and LDL-C, with thresholds >225–235 ng/mL indicating high vascular risk. However, its association may partly depend on oxidized LDL levels, positioning it as a marker of oxidative stress within coronary inflammation rather than a fully independent causal factor [122].

A 2025 meta-analysis of 22 studies (1110 stable vs. 1298 unstable plaques) reported a pooled sensitivity of 85% (95% CI: 80–89%), specificity of 80% (95% CI: 74–85%), and AUC of 0.89 (95% CI: 0.86–0.92), indicating robust performance across vascular sites including coronaries. The positive likelihood ratio (PLR) was 4.23, and the diagnostic odds ratio (DOR) was 22.55, supporting its utility for plaque vulnerability assessment [123]. In coronary CT angiography contexts, Lp-PLA2 at cutoffs like 183 ng/mL showed 91.7% sensitivity for detecting plaques or stenosis, outperforming some imaging markers alone. However, for event prediction in asymptomatic cohorts, accuracy drops (e.g., 37% sensitivity, 75% specificity in the top quartile). These findings highlight better strength for plaque characterization than primary screening. Heterogeneity in plaque criteria and location affects results, but overall, an AUC > 0.85 confirms good discriminative power. MPO and Lp-PLA2 both detect plaque inflammation but describe different mechanisms, with MPO emphasizing acute oxidative neutrophil activity and Lp-PLA2 targeting chronic macrophage-driven lipid hydrolysis. Neither of these markers has head-to-head superiority for detection alone, but combinations enhance accuracy.

Myeloperoxidase (MPO) acts as a marker of coronary vascular inflammation by reflecting neutrophil activation and oxidative stress within atherosclerotic plaques. Released from neutrophils in inflamed coronary lesions, it promotes LDL oxidation, endothelial dysfunction, and plaque instability. High MPO (>320 pmol/L) predicts major adverse cardiac events in ACS patients, with meta-analyses showing odds ratios up to 3.5 for mortality, complementing troponins for short-term risk stratification. It identifies high-risk patients even with low hsCRP. Consequently, MPO levels highlight plaque-specific oxidative inflammation over general markers [124].

Soluble CD40 ligand (sCD40L) and pentraxin-3 (PTX3) provide improved specificity for endothelial activation and local plaque inflammation compared to hsCRP.

SCD40L serves as a marker of coronary inflammation by mediating interactions between activated platelets, endothelial cells, and immune cells in atherosclerotic plaques, promoting cytokine release, matrix metalloproteinase expression, and plaque instability. Its primary source is from activated platelets during thrombosis (>95%). Levels rise significantly in ACS (e.g., mean 68 ng/mL in STEMI vs. <30 ng/mL in controls), reflecting acute plaque rupture and inflammation intensity.

ROC analysis shows an AUC 0.632 for ACS prediction (cutoff 5.68 ng/mL; sensitivity 82%, specificity 40%), with stronger performance for STEMI over NSTEMI/UA [125]. Elevated sCD40L independently predicts adverse events like recurrent MI and revascularization, though antiplatelet therapy may blunt its levels. sCD40L, MPO, and Lp-PLA2 all mark coronary inflammation but differ in pathways, timing, and performance, with MPO and Lp-PLA2 generally outperforming sCD40L in predictive strength for plaque vulnerability.

MPO and Lp-PLA2 show superior AUC and independence from hsCRP for event prediction, while sCD40L adds value in thrombotic ACS subsets. Combinations (e.g., all three) may optimize detection, though direct trials are lacking.

Pentraxin 3 (PTX3) emerges as a promising future biomarker for detecting vascular inflammation in coronary artery disease (CAD), offering greater specificity than hsCRP or IL-6 by localizing primarily to atherosclerotic lesions rather than reflecting systemic inflammation. PTX3 acts as a soluble pattern recognition receptor rapidly produced at sites of inflammation, such as in coronary plaques, enabling earlier detection of subclinical atherosclerosis. Unlike hsCRP, which is a downstream acute-phase reactant influenced by multiple factors, PTX3 correlates directly with plaque instability and cardiovascular events independent of traditional risk factors. Clinical studies show elevated PTX3 levels predict CAD progression and all-cause mortality in older adults [126].

PTX3 localizes to atherosclerotic plaques as a rapid-response pattern recognition molecule, directly reflecting lesion inflammation and instability rather than broad systemic effects. Studies link elevated PTX3 to CAD progression and mortality, independent of CRP [127].

TRAF3IP2, an IL-17 pathway adaptor, shows upregulated expression in CAD, enabling nomogram-based prediction of stenosis severity via bioinformatics and cohort validation. It bridges inflammation to clinical outcomes more precisely than cytokines [128].

Several biomarkers show promise in distinguishing stable from unstable coronary plaques by reflecting plaque-specific inflammation, matrix degradation, or immune infiltration rather than systemic effects. These include sLOX-1, MMP-9, HSPA2, GEM, and MPO, validated through bioinformatics, OCT imaging, and cohort studies. SLOX-1 and MMP-9 levels are elevated in ruptured plaques, with a combinatorial model (including WBC and CK-MB) achieving moderate sensitivity (62%) and high specificity (98%) for rupture detection in ACS patients via OCT. HSPA2 and GEM, identified as differentially expressed genes, exhibit strong diagnostic AUCs (0.93 and 0.91 in training cohorts) and downregulation in unstable plaques, confirmed by immunohistochemistry [129].

HSPA2 and GEM stand out as protein biomarkers with the highest reported AUC values for distinguishing unstable coronary plaques, based on bioinformatics and machine learning analyses of plaque gene expression. These markers show strong diagnostic performance, with HSPA2 achieving AUC 0.934 in training cohorts and 0.828 in validation, while GEM reaches 0.913 and 0.788, respectively [130].

HSPA2 (a heat shock protein) and GEM (a GTP-binding protein) are downregulated in unstable plaques and correlate with immune cell infiltration patterns like macrophages and neutrophils. Their combined use enhances specificity for plaque instability over systemic markers.

Circulating proteins like soluble LOX-1 (sLOX-1), pregnancy-associated plasma protein A (PAPP-A), and myeloperoxidase (MPO) outperform troponin in predicting plaque instability by detecting early vascular inflammation and rupture before myocardial necrosis occurs [131]. sLOX-1 rises earlier post-ACS than troponin-T, reflecting oxidized LDL uptake in plaques and enabling detection of pre-necrotic instability in prospective cohorts. PAPP-A predicts outcomes in troponin-negative ACS patients with high hazard ratios (up to 7.48), signaling matrix degradation in vulnerable plaques. MPO correlates directly with ongoing rupture in ACS, distinguishing acute instability from stable states better than troponin or CRP.

Plasma proteomics platforms, particularly those using Olink Proximity Extension Assay (PEA) technology, report the highest AUCs for plaque instability markers by analyzing hundreds of circulating proteins simultaneously. Olink PEA panels achieved AUC 0.855 for predicting accelerated plaque progression, surpassing clinical models (ΔAUC 0.137, *p* = 0.004) in CCTA-validated cohorts. Targeted proteomics via mass spectrometry or aptamer-based SomaScan also yield high AUCs (0.80–0.93) for high-risk plaques, identifying signatures like FRIL, CATD, and MMP9 [132].

MicroRNAs (e.g., miR-133a-3p, miR-451a) show promise as upregulated markers in CAD but require validation. These miRNAs are upregulated in CAD plasma, potentially from cardiomyocyte damage or endothelial dysfunction in inflamed coronary plaques. MiR-133a-3p regulates cardiac remodeling and inflammation pathways, while miR-451a links to vascular smooth muscle proliferation and oxidative stress in atherosclerosis. Their stability in circulation positions them as non-invasive indicators of ongoing coronary pathology beyond acute injury. ROC analyses highlight miR-133a-3p and miR-451a as top performers among panels (e.g., with miR-21-5p, miR-221-3p), showing strong discriminatory power for CAD diagnosis versus controls, though specific AUC values and inflammation cutoffs require further validation in larger cohorts. They complement protein markers like MPO by capturing genetic-regulatory aspects of plaque vulnerability. Systematic reviews identify miR-133a-3p as specifically upregulated in ACS versus stable CAD, alongside miR-1, miR-208a/b, and miR-499, due to its cardiac-specific release during acute necrosis and inflammation. In stable CAD cohorts, levels remain closer to controls or show less elevation, as plaque rupture and thrombosis—hallmarks of ACS—are absent [133]. A key meta-analysis of 9 studies reported a pooled AUC of 0.73 (95% CI: 0.68–0.79) for miR-133a-3p in ACS contexts, rising to 0.89 with healthy controls versus 0.68 with symptomatic ones. Although sensitivity and specificity were not uniformly pooled, they were concordant with the high values found in early studies (e.g., >0.86) [134].

Computed tomography angiography (CCTA) with perivascular fat attenuation index (FAI) directly quantifies coronary inflammation by measuring fat changes around proximal coronary arteries, predicting events independently of hsCRP. Positron emission tomography (PET-CT) using tracers like ^18^F-FDG targets metabolically active inflamed plaques in coronary vessels with high specificity. These outperform blood markers for localizing inflammation to the coronary circulation [135].

FAI quantifies the shift from lipid-rich (low attenuation) to water-rich (higher attenuation) perivascular adipose tissue (PVAT) caused by paracrine inflammatory signals from the adjacent coronary wall, acting as a “vessel wall thermometer” specific to local vascular inflammation. This spatial gradient around proximal major coronaries (e.g., right coronary artery, left anterior descending) isolates coronary-specific effects, avoiding systemic confounders like infections or obesity that affect hsCRP. FAI predicts plaque instability, hemodynamic impact (e.g., lower CT-FFR), and cardiac events independently of traditional risk scores or hsCRP, enabling risk reclassification in up to 62% of patients. It dynamically responds to anti-inflammatory therapies (e.g., biologics reducing FAI in months), confirming its link to treatable coronary processes. Cutoffs like ≥−70.1 HU signal high-risk inflammation linked to plaque rupture [136].

An ideal marker for detecting inflammation in ACS would offer high specificity to vascular or plaque inflammation, rise early before necrosis (unlike troponin), predict instability and outcomes independently, and enable point-of-care testing. It must localize to atherosclerotic lesions, distinguishing ACS from non-cardiac inflammation, with rapid kinetics (peaks within hours) and minimal circadian variation, unlike IL-6. High sensitivity/specificity (AUC > 0.90) for risk stratification complements hs-cTn by identifying pre-rupture events in troponin-negative patients. The marker should predict instability in troponin-negative patients, guide anti-inflammatory therapies such as colchicine, and improve net reclassification over hsCRP by integrating with imaging to assess plaque vulnerability.

The future of inflammatory markers in acute coronary syndrome (ACS) holds multi-omic panels integrating proteomics, transcriptomics, and AI-driven models, offering superior risk stratification beyond single markers such as hsCRP.

Olink PEA and SomaScan platforms evolve into point-of-care multiplex assays, integrating machine learning (e.g., CatBoost) to achieve AUC > 0.85 for plaque progression, validated against CCTA/OCT. MicroRNA-mRNA networks and deep learning forecast personalized therapies like IL-1β inhibitors [137].

## 5. Therapeutic Strategies and Implications

### 5.1. Aspirin

Aspirin plays a significant anti-inflammatory role in ACS, beyond its well-known antiplatelet effects. In ACS, vascular injury involves complex interactions among inflammatory mediators, platelets, and thrombosis. Aspirin at antiplatelet doses (100–300 mg/day) reduces inflammation markers such as CRP or TNF-α [138], macrophage colony-stimulating factor (M-CSF), and monocyte chemoattractant protein-1 (MCP-1). Aspirin exerts immunomodulatory effects by counteracting the increase in pro-inflammatory monocyte subsets (e.g., CD14^high^CD16^+^ monocytes) seen in ACS. This suggests aspirin can reduce monocytic biomarkers of inflammation, further contributing to plaque stabilization and improved outcomes [139]. Additionally, aspirin may acetylate COX-2 in vascular cells, shifting its activity to produce 15-epi-lipoxin (“aspirin-triggered lipoxin”), a potent anti-inflammatory mediator. Aspirin also stimulates endothelial nitric oxide synthase (eNOS) and upregulates heme oxygenase-1 (HO-1), enhancing the antioxidative capacity of vascular cells [140]. Mechanistically, aspirin inhibits platelet cyclooxygenase-1 (COX-1), thereby reducing thromboxane A2 formation. This not only prevents platelet aggregation but also decreases thrombin formation and the production of secondary pro-inflammatory mediators, such as sphingosine-1-phosphate. This inhibition helps break the cycle of platelet activation and inflammation that contributes to plaque instability and thrombosis [138]. Aspirin may help decrease the progression of atherosclerosis by protecting LDL from oxidative modification and improving endothelial dysfunction. When LDL is exposed to oxidative stress, aspirin treatment significantly decreases the production of malondialdehyde (a marker of lipid peroxidation) and diminishes the electrophoretic mobility of LDL, both indicators of reduced oxidative modification. These effects are believed to reduce vascular inflammation further and enhance vasodilation. The protective effect is thought to be related to aspirin’s ability to inhibit the formation of free radicals and lipid peroxides that drive LDL oxidation [141,142]. The ability of aspirin to protect LDL from oxidative modification is dose-dependent, with higher doses showing protective effects (300 mg/day) and lower doses (75 mg/day) potentially having the opposite effect [143]. Aspirin’s anti-inflammatory effects in ACS are achieved primarily through inhibition of COX enzymes, leading to reduced production of inflammatory and prothrombotic mediators, modulation of immune cell activity, and improvement of endothelial function. These mechanisms collectively reduce inflammation, stabilize plaques, and lower the risk of adverse cardiovascular events [144].

While low-dose aspirin (75–100 mg/day) is effective for antiplatelet therapy and cardiovascular prevention, its effect on LDL oxidation may not be beneficial. It could even be pro-oxidative at these doses. The antioxidant, LDL-protective effects of aspirin are observed at higher doses (300 mg/day). Still, these are not typically used for long-term cardiovascular prevention due to the increased risk of side effects and the exceptionally high risk of gastrointestinal bleeding [143,145].

### 5.2. Statins

Statins have a significant anti-inflammatory effect in ACS, in addition to their well-known lipid-lowering properties.

The JUPITER trial was a large, randomized, double-blind, placebo-controlled trial involving 17,802 participants who were healthy but had low levels of LDL cholesterol (the “bad” cholesterol) and high levels of CRP (>2 mg/L). Participants were assigned to receive either rosuvastatin or a placebo and were followed for an average of 1.9 years. Rosuvastatin reduced LDL cholesterol levels by 50% and CRP levels by 37%, indicating its effectiveness in lowering both cholesterol and inflammation. The findings suggest that statin therapy, particularly with rosuvastatin, could be beneficial for individuals who do not have high cholesterol but do have elevated CRP levels, which is often associated with a higher risk of cardiovascular issues [146]. Statins inhibit nuclear factor kappa B (NF-κB), a key transcription factor that regulates the expression of many inflammatory genes, thereby reducing inflammatory markers such as IL-1β, CRP, IL-6, and TNF-α [147]. Stabilization of atherosclerotic plaques by reducing inflammation within atherosclerotic plaques, making them less likely to rupture and cause acute events, partly due to reduced infiltration of inflammatory cells (like macrophages) and decreased expression of adhesion molecules. Statins reduce the expression of adhesion molecules on endothelial cells (e.g., ICAM-1, lymphocyte function-associated antigen-1) and chemokines such as monocyte chemoattractant protein-1 (MCP-1). This inhibits the transendothelial migration of leukocytes into the vessel wall, limiting inflammatory cell infiltration into atherosclerotic plaques [148].

Additionally, statins reduce Myeloperoxidase (MPO) activity, decrease oxidative stress and vascular inflammation, and improve endothelial function by enhancing nitric oxide bioavailability, which reduces endothelial activation and inflammation [149].

Statins can reduce inflammatory markers within days of starting therapy, even before significant changes in cholesterol levels occur. This rapid effect is particularly beneficial in the acute phase of ACS [150].

Different types of statins vary in their anti-inflammatory effects in ACS and other cardiovascular conditions, influenced by their chemical properties (lipophilicity vs. hydrophilicity), potency, and dosage. Rosuvastatin and atorvastatin, both potent statins, significantly reduce CRP levels. Lipophilic statins (e.g., simvastatin, atorvastatin) tend to have more pleiotropic effects, including anti-inflammatory actions, compared to hydrophilic statins (e.g., pravastatin, rosuvastatin) [151]. Some evidence suggests rosuvastatin may paradoxically increase certain pro-inflammatory cytokines (e.g., IL-1β) in specific contexts, although it still reduces other markers like IL-6 [152]. However, one study reported that rosuvastatin (20 mg) reduced CRP levels greater than atorvastatin (40 mg; 44 vs. 35%) after 4 weeks of treatment, indicating a more potent anti-inflammatory effect for rosuvastatin [153]. In conclusion, while all statins share anti-inflammatory properties, lipophilic statins like simvastatin and atorvastatin often exhibit stronger anti-inflammatory effects, possibly due to better cellular penetration. Rosuvastatin, a hydrophilic statin, shows potent CRP reduction but may have a mixed impact on other cytokines. The intensity of anti-inflammatory action also depends on the statin dose, with higher doses generally producing greater effects.

In the MIRACLE study, atorvastatin 80 mg in patients with ACS (NSTEMI and UA) reduced the rate of ischemic cardiac events over a 16-week follow-up period, with the effect already visible after 6 weeks [154]. High-dose statin (atorvastatin 80 mg) was also shown to be superior to pravastatin 40 mg in patients with ACS in the PROVE IT-TIMI 22 trial, reducing primary and secondary endpoint (death, myocardial infarction, unstable angina requiring rehospitalization, stroke, or revascularization ≥ 30 days) [155].

In registries that included the follow-up of patients with ACS, high-dose statin administration within the first 24 h of first medical contact reduced the incidence of MACE and the risk of in-hospital mortality [156]. A Cochrane review of over 14,000 ACS patients found no significant increase in serious adverse events with early statin initiation (within 14 days of ACS onset) compared to placebo or usual care [157,158].

### 5.3. PCSK9 Inhibitors

PCSK9 inhibitors have a notable anti-inflammatory role in ACS that extends beyond their potent effects on lowering LDL cholesterol. Emerging evidence from clinical and experimental studies highlights several mechanisms by which PCSK9 inhibition modulates inflammation and vascular health in ACS. PCSK9 levels are significantly elevated during ACS and are associated with increased expression of pro-inflammatory cytokines such as TNF-α, IL-6, IL-1β, IFN-γ, and chemokines like MCP-1. Higher PCSK9 correlates with systemic inflammatory markers, including white blood cell count (WBC), fibrinogen, and high-sensitivity C-reactive protein (hs-CRP). PCSK9 promotes vascular inflammation by enhancing monocyte recruitment, endothelial adhesion molecule expression (e.g., ICAM-1), and the activation of inflammatory cascades, thereby contributing to plaque progression and instability [159]. In the ATHEROREMO-IVUS study, serum PCSK9 levels were correlated with the necrotic core size of atherosclerotic plaques in patients with ACS, independent on the LDL-cholesterol values [160]. Hence, PCSK9 inhibitor administration in patients with acute coronary syndromes may have specific anti-inflammatory effects that occur in addition to lowering LDLc. Anti-inflammatory mechanisms of PCSK9 inhibitors are to reduce the expression of proinflammatory cytokines such as TNF-α, IL-1β, IL-6 and MCP-1, both in animal models and in human patients [157,160], to modulate immune activation by favoring dendritic cell maturation and activation of T lymphocytes towards a proinflammatory phenotype, to reduce monocyte migration by decreasing the expression of CCR2 and ICAM-1, reduce the activation of inflammatory pathways such as TLR4/NF-κB and LOX-1/NF-κB, leading to reduced expression of proinflammatory genes and increased anti-inflammatory cytokines (e.g., IL-10) [161].

Several meta-analyses and randomized clinical trials have shown that although PCSK9 inhibitors dramatically lower LDL cholesterol, there is no significant decrease in hsCRP compared with placebo or other therapies [157,162,163]. For example, a meta-analysis of 4198 participants showed a minimal and statistically insignificant reduction in hsCRP (−0.04 mg/L) [164]. Similar results have been reported in studies such as EQUATOR [165] and SPIR [166], where the addition of PCSK9 inhibitors to statin therapy did not influence hsCRP levels, despite substantial reductions in LDL-C. We can say that the anti-inflammatory effect of PCSK9 inhibitors is not reflected and cannot be measured by hsPCR. Still, their effect is related to atheroma plaque activity rather than to changes in circulating levels of acute phase reactants. Patients treated with PCSK9 inhibitors had a reduced inflammatory load in carotid atherosclerotic plaques, characterized by lower levels of NLRP3, caspase-1, IL-1β, and TNF-α in carotid plaques, even in subgroups with LDL-C levels below 100 mg/dL [167].

### 5.4. Ezetimibe

Administration of ezetimibe in combination with statins (rosuvastatin or pitavastatin) in patients with acute coronary syndrome (ACS) resulted in significant decreases in inflammatory markers, such as sPLA2-IIa and interleukin-1β, compared to statin therapy alone. A more minor increase in endothelial adhesion molecules (VCAM-1 and ICAM-1) was also observed in the combination therapy group, suggesting an additional protective effect on the endothelium of the vasculature [168]. Some studies have shown that the anti-inflammatory and cardiovascular protective benefits of ezetimibe cannot be attributed solely to LDL-C reduction. Anti-inflammatory effects, reduction in oxidative stress, and myocardial fibrosis may contribute to a decrease in cardiovascular events and hospitalizations for heart failure after ACS [169]. Data from the IMPROVE-IT trial, which included more than 18,000 patients with ACS, show that the addition of ezetimibe to statin therapy (simvastatin) resulted in a further reduction in hsCRP of about 16% at one month and 14% throughout the study compared with statin monotherapy. More patients treated with ezetimibe/simvastatin achieved the target hsCRP < 2 mg/L compared with those treated with simvastatin alone (50% vs. 29%, *p* < 0.001) [170]. In the ENHANCE trial, the addition of ezetimibe to simvastatin doubled the reduction in hsCRP (49.2% vs. 23.5% with statin alone). However, the decrease in LDL-C was similar between groups. This result suggests an additional mechanism, possibly independent of LDL-C [171].

### 5.5. Inhibition of NLRP3 Inflammasome in ACS

Inflammasome NLRP3 (NOD-, LRR- and Pyrin domain-containing protein 3) is an essential cytosolic multiprotein complex of the innate immune system involved in the regulation of inflammation and immune response. NLRP3 inflammasome is a key mediator of sterile inflammation in ACS, linking atherosclerotic plaque destabilization to myocardial injury and adverse remodeling. Its inhibition represents a promising strategy for secondary prevention, with strong evidence from preclinical and clinical studies [172,173,174].

### 5.6. MCC950

MCC950 is a selective and potent inhibitor of the NLRP3 inflammasome, with promising results in preclinical models of ACS. Administration of MCC950 to mice with myocardial infarction resulted in reduced myocardial fibrosis and improved cardiac function compared with untreated animals [175]. In models of cerebral ischemia/reperfusion (analogous to myocardial reperfusion injury), MCC950 significantly reduced infarct size, edema, and markers of apoptosis, suggesting similar protective potential at the cardiac level as well. This effect is due to the inhibition of caspase-1 and IL-1β release, thereby reducing inflammatory cell death (pyoptosis) [176].

For now, the evidence comes from preclinical animal studies; there are no human clinical data yet on MCC950 in ACS.

### 5.7. OLT1177

The administration of OLT1177 (dapansutrile) in acute coronary syndromes (ACS) has a promising role as a targeted anti-inflammatory therapy by selectively inhibiting the NLRP3 inflammasome. OLT1177 directly blocks the activation and oligomerization of NLRP3, preventing its interaction with ASC and caspase-1, leading to reduced maturation and release of the proinflammatory cytokines IL-1β and IL-18 [16,96]. It does not affect other inflammasomes (AIM2, NLRC4), which gives it specificity and a superior safety profile [177]. Administration of OLT1177 to animals with myocardial ischemia/reperfusion significantly reduced infarct size (by 36–71%, dose-dependently) and maintained left ventricular systolic function at 24 h and 7 days after injury [178]. OLT1177 reduces the levels of IL-1β, IL-18, IL-6, and other pro-inflammatory cytokines in both tissues and circulation, thereby limiting neutrophil infiltration and oxidative stress. It improves cardiac muscle oxidative metabolism and reduces proinflammatory metabolic markers [179]. OLT1177 is well-tolerated in humans, with no significant adverse effects or biochemical or hematologic changes observed at high doses [177]. Early phase clinical trials have shown benefit in patients with heart failure and gout, and development for cardiovascular indications is ongoing [17].

### 5.8. Colchicine

Colchicine blocks the assembly of the NLRP3 inflammasome, thereby reducing its activation and the release of proinflammatory cytokines such as interleukin-1β (IL-1β) and IL-18, key molecules in amplifying the inflammatory response after myocardial infarction. Colchicine binds to tubulin and destabilizes microtubules, thereby inhibiting microtubule-dependent functions of inflammatory cells (neutrophils, monocytes/macrophages). This reduces the migration, activation and release of inflammatory mediators by these cells. Colchicine inhibits neutrophil chemotaxis and activation, as well as the formation of neutrophil extracellular traps (NETs), which contribute to tissue injury and thrombosis progression in ACS [180,181].

Results from the Colchicine Cardiovascular Outcomes Trial (COLCOT) show that colchicine (0.5 mg/day) in patients with recent MI (about 14 days after the MI) significantly reduces the risk of major ischemic cardiovascular events compared with placebo in addition to standard treatment. The risk reduction was mainly driven by a decrease in the incidence of stroke and urgent revascularization. No significant differences were observed between groups in cardiovascular mortality or recurrent MI (HR = 0.77 (95% CI: 0.61–0.96), *p* = 0.02 reduction in the risk of major cardiovascular events) [182].

The study did not include detailed measurements of inflammatory biomarkers at baseline and throughout the study, making it difficult to correlate the inflammatory response with the observed clinical benefits directly. The COLCOT did not demonstrate a significant reduction in total or cardiovascular mortality, but only in composite events, and the number of deaths was almost identical between groups (43 vs. 44) [183].

Results from the COPS (Colchicine in Patients With Acute Coronary Syndrome) trial of colchicine in patients with ACS showed that at 12 months, colchicine did not significantly reduce the risk of major cardiovascular events in patients with ACS (24 events in the colchicine group vs. 38 in the placebo group, *p* = 0.09; HR 0.65), but was associated with an increase in total mortality (8 deaths in the colchicine group vs. 1 in the placebo group, HR 8.20; *p* = 0.047), mainly driven by increased non-cardiovascular mortality (5 vs. 0, *p* = 0.024). In the COPS study, colchicine was administered very early, during hospitalization for ACS, before discharge, immediately after the diagnosis of the acute coronary event. The administration schedule was as follows: 0.5 mg twice daily (1 mg/day) for the first month, followed by 0.5 mg once daily for the next 11 months [181].

The increase in non-cardiovascular mortality observed in the COPS study in patients treated with colchicine has no apparent cause and remains controversial. Detailed analysis showed that four of the five non-cardiovascular deaths were related to sepsis. Still, three of these cases occurred in patients who had discontinued colchicine at least 30 days before death, so were not on active treatment at the time of the event. Another death was caused by cancer, with no known link to colchicine [180]. Colchicine increases the risk of gastrointestinal events and possibly infections, but without a clear association with increased non-cardiovascular mortality on a large scale [184].

Administration of colchicine for 12 months after ACS significantly reduced major cardiovascular events at 24 months, mainly due to a decrease in urgent revascularizations (colchicine 3 cases vs. placebo 16 cases, HR 0.19; 95% CI: 0.05–0.66; *p* = 0.009), without significantly influencing total mortality (colchicine 9 deaths vs. placebo 4 deaths, statistically insignificant difference (HR 2.28; 95% CI: 0.7–7.4; *p* = 0.17) or non-cardiovascular mortality (colchicine 5 deaths vs. placebo 2 deaths, statistically negligible difference (HR 2.54; 95% CI: 0.49–13; *p* = 0.27) [185].

A meta-analysis that included only two trials (COLCOT and COPS) showed that colchicine administration in ACS reduces the risk of MACE (composite events: cardiovascular death, recurrent myocardial infarction, stroke or urgent revascularization) by 25–30%. This effect is primarily driven by a decrease in stroke rates (48–52% reduction) and a reduction in coronary revascularization requirements (23–32% reduction) [184]. With just two randomized controlled trials (RCTs) in the review, the findings might not represent broader clinical experiences. A larger number of studies would provide more confidence in the reliability of the results. Even though both trials investigated the use of colchicine for patients with acute coronary syndrome, there were variations in how the drug was administered. For example, one study used a higher initial dosing regimen than the other. These differences might have affected the outcomes slightly and can limit how broadly the conclusions from the meta-analysis can be applied.

Additionally, the timing of administration of colchicine after an ACS is essential. A sub-analysis of the COLCOT study showed that early treatment, specifically within the first three days after the heart attack, was shown to substantially lower the chance of experiencing a composite event that included heart-related death, another heart attack, stroke, resuscitated cardiac arrest, or the need for urgent hospital procedures like revascularization. The results indicate that initiating treatment in the hospital, rather than waiting until after discharge, can lead to significantly better outcomes for patients [186].

Other large trials, such as CLEAR SYNERGY [187], have not confirmed the benefits observed in COLCOT, suggesting that the effect of colchicine may be more modest or dependent on population selection and timing of initiation of therapy. Results from the CLEAR SYNERGY (OASIS 9) study show that colchicine in patients with acute MI treated with coronary angioplasty (PCI) did not significantly reduce the risk of major adverse cardiovascular events (MACE) compared with placebo after a median follow-up of almost 3 years. In the CLEAR SYNERGY trial, colchicine was administered to patients with acute myocardial infarction (both STEMI and extended NSTEMI) as soon as possible after percutaneous coronary intervention (PCI), but no later than 72 h after PCI [188]. The CLEAR SYNERGY trial demonstrates that, in patients with acute MI treated by PCI, routine colchicine administration does not reduce MACE compared with placebo (cardiovascular death: 3.3% colchicine vs. 3.2% placebo (HR 1.03); death from any cause: 4.6% colchicine vs. 5.1% placebo (HR 0.90); recurrent MI: 2.9% colchicine vs. 3.1% placebo (HR 0.88); ischemia-driven revascularization: 4.6% colchicine vs. 4.7% placebo (HR 1.01), although it reduces inflammatory markers (hsCRP at 3 months, colchicine: 2.98 mg/dL vs. placebo: 4.27 mg/dL (*p* < 0.001)—significant decrease in inflammation, without substantial clinical impact).

In the colchicine-treated group, there was a higher incidence of gastrointestinal side effects compared with placebo (diarrhea: 10.2% colchicine vs. 6.6% placebo (*p* < 0.001)), without an increase in the number of infections (serious infections: 2.5% colchicine vs. 2.9% placebo (*p* = 0.85)) [187].

Possible explanations between the results of COLCOT and CLEAR SYNERGY would be the very early randomization in patients with STEMI and severe inflammation (CLEAR SYNERGY included more than 7000 patients, mostly with STEMI, randomized very early (on average 1.6 h after PCI, but maximum 72 h post-infarction), insufficient inflammatory control (In CLEAR SYNERGY, levels of C-reactive protein (CRP), a marker of inflammation, remained elevated at 3 months (mean 3.0 mg/L in colchicine vs. 4.3 mg/L in placebo), in contrast to COLCOT, where CRP fell below 2 mg/L), the impact of the COVID-19 pandemic (a significant part of the CLEAR SYNERGY study was conducted during the COVID-19 pandemic, which disrupted health systems and led to underreporting of non-fatal events [heart attacks, revascularizations]), on event reporting and patient care, differences in design and statistical power. CLEAR SYNERGY had more than twice as many events as COLCOT, which may suggest that the positive effect in smaller trials may be overestimated (regression to the mean; in COLCOT, the benefit was driven mainly by reduction in emergency hospitalizations for angina and revascularization, not so much by reduction in deaths or major MIs) [189].

These elements suggest that the benefits of colchicine observed in COLCOT cannot be automatically generalized to all myocardial infarction populations, and its efficacy depends on the clinical context and timing of administration.

### 5.9. Interleukin-1β Inhibitors

Inhibition of the IL-1β pathway may play a crucial role in the treatment of ACS by modulating the inflammatory response with beneficial effects on both acute and long-term cardiac remodeling.

The pivotal study evaluating canakinumab (an anti-IL-1β monoclonal antibody) in ACS is the Canakinumab Anti-inflammatory Thrombosis Outcomes Study (CANTOS). It included 10,061 patients with a history of myocardial infarction and elevated ultrasensitive C-reactive protein (hsCRP ≥ 2 mg/L) who received canakinumab (50 mg, 150 mg or 300 mg) or placebo, administered subcutaneously every 3 months, with a median follow-up of 3.7 years [190].

Canakinumab reduced not only the first major event, but also the total number of serious cardiovascular events (including coronary revascularization), with a relative reduction of about 20% for all doses tested. Canakinumab therapy significantly decreased hsCRP levels compared with placebo, with a greater reduction at higher doses (*p* < 0.001 for all doses). Secondary analyses showed that patients who achieved hsCRP < 2 mg/L after 3 months of treatment had a 25% reduction in major cardiovascular events, a 31% decrease in cardiovascular mortality, and a similar reduction in total mortality. Canakinumab was associated with an increased risk of fatal infections or sepsis (0.31/100 patient-years vs. 0.18/100 patient-years for placebo), but the overall rate of serious adverse events was comparable to placebo.

CANTOS demonstrated for the first time that targeted inhibition of inflammation (by blocking IL-1β) reduces the risk of recurrent cardiovascular events in patients with previous myocardial infarction and residual inflammation, independent of cholesterol lowering.

The effect was more pronounced in responding patients with reduced inflammation (hsCRP < 2 mg/L). Wide clinical use of canakinumab is limited by cost and risk of infection, but the study validates the concept of anti-inflammatory therapy in secondary prevention after ACS [191].

It should be noted that despite the benefits shown in CANTOS, canakinumab has not been imposed in the treatment of patients with ACS due to the increased risk of serious infections (in the CANTOS, the rate of fatal infections was almost double that of placebo), very high costs (over 50,000 USD/year, making it inaccessible for widespread use in ACS) [192], the lack of a formal indication for this pathology (it is only approved for rare auto-inflammatory conditions [e.g., cryopyrin-associated periodic syndromes—CAPS, juvenile systemic arthritis, not for ACS]) and the existence of safer and more affordable alternatives such as colchicine [190].

Anakinra, an IL-1 receptor antagonist, has a promising role in ACS, acting by inhibiting the inflammatory response that contributes to myocardial injury and adverse ventricular remodeling after infarction. The administration of anakinra in patients with STEMI (VCUART3—Virginia Commonwealth University Anakinra Remodeling Trial 3) significantly reduced hsCRP levels compared to placebo (both standard and high dose) during the first 14 days (median 67 IQR 39–120 vs. 214 IQR 131–394 mg-day/L; *p* < 0.001) [193]. There was no significant difference between the standard (once-daily) and high-dose (twice-daily) anakinra treatments in reducing inflammation. This finding suggests that the standard dose is adequate to block the interleukin-1 receptor, thereby modulating the inflammatory response after STEMI. Notably, the trial found a lower incidence of heart failure events, such as hospitalizations and new-onset heart failure, in patients treated with anakinra (9.4%) compared to those treated with placebo (25.7%) (*p* = 0.046). This suggests that targeting the interleukin-1 pathway with anakinra may offer benefits in reducing the risk of complications, such as heart failure, following STEMI.

There were no significant differences in the incidence of severe infections between the anakinra and placebo groups (14% vs. 14%). Injection site reactions were more common with anakinra. No significant differences were noted in the interval changes in left ventricular end-systolic volume or ejection fraction between the anakinra-treated groups and the placebo group. This implies that while anakinra effectively dampens inflammation, it does not significantly alter the structural remodeling of the heart within the measured time frame.

### 5.10. Low-Dose Interleukin-2 (IL-2)

In the context of acute coronary syndrome (ACS), the administration of low-dose interleukin-2 (IL-2) serves primarily as an immunomodulatory intervention designed to mitigate vascular inflammation through the expansion of regulatory T cells (Tregs). These cells possess significant immune-suppressive and anti-inflammatory properties. At low doses, IL-2 selectively enhances the quantity and functionality of Tregs without markedly increasing effector T cells. Tregs play a crucial role in suppressing the inflammatory processes that contribute to atherosclerosis and acute coronary events. In ACS, vascular inflammation is a principal factor in disease progression and adverse cardiovascular outcomes. By promoting the expansion of Tregs, low-dose IL-2 effectively reduces this inflammation, potentially stabilizing atherosclerotic plaques and enhancing vascular health [194].

The LILACS trial established that low-dose IL-2 is both safe and biologically effective for patients with acute coronary syndrome (ACS) and stable ischemic heart disease. The study demonstrated an increase in regulatory T cells (Tregs) without any serious adverse events. The target dose required to achieve a significant increase in Tregs was approximately 1.46 × 10^6^ IU [195].

The IVORY trial, a randomized, placebo-controlled study, demonstrated that low-dose IL-2 administration in patients with acute coronary syndrome (ACS) resulted in a significant reduction in arterial inflammation, as assessed by ^18^F-FDG PET/CT imaging. This outcome was correlated with a notable increase in circulating regulatory T cells (Tregs) and no increase in effector T cells. The treatment was well tolerated. A trend toward fewer major adverse cardiovascular events (MACE) was observed in the IL-2-treated group over a median follow-up period of 2.5 years, indicating potential clinical benefit; however, larger trials are necessary to confirm these findings [194].

Low-dose IL-2 therapy in ACS acts by expanding regulatory T cells, thereby reducing vascular inflammation and potentially lowering the risk of subsequent cardiovascular events. It represents a promising targeted immunomodulatory approach to improve outcomes in high-risk ACS patients with an acceptable safety profile demonstrated in early-phase clinical trials.

### 5.11. IL-6 Inhibition

Interleukin-6 inhibitors are emerging as potential therapeutic agents in ACS due to IL-6′s central role in propagating inflammation, activating leukocytes and adipose tissue to amplify inflammation, stimulating hepatic production of hs-CRP (a biomarker of cardiovascular risk), promoting endothelial dysfunction, plaque instability, and adverse remodeling post-ACS [196].

Currently, there are no medications specifically approved for targeting IL-6 in acute coronary syndrome (ACS). However, some IL-6 inhibitors approved for other conditions have been studied in ACS with promising but still investigational results [197].

Tocilizumab is a monoclonal antibody against the IL-6 receptor, is approved by the FDA for rheumatoid arthritis, juvenile idiopathic arthritis, and giant cell arteritis [198]. Tocilizumab, an interleukin-6 receptor (IL-6R) antagonist, has been investigated as a potential therapy for acute coronary syndrome (ACS), particularly acute myocardial infarction (AMI), due to its anti-inflammatory properties and its ability to modulate the inflammatory response that contributes to myocardial injury and adverse outcomes. In the randomized, double-blind, placebo-controlled ASSAIL-MI trial [199], patients presenting with STEMI within 6 h of symptom onset received a single intravenous dose of tocilizumab (280 mg) or placebo before PCI. Tocilizumab significantly increased the myocardial salvage index compared to placebo (adjusted between-group difference 5.6 percentage points, *p* = 0.04). There was also less microvascular obstruction in the tocilizumab group, although the difference in final infarct size did not reach statistical significance. Adverse events were similar between groups, and the drug was well tolerated. The results suggest that tocilizumab may reduce reperfusion injury and enhance myocardial salvage in STEMI patients, likely by attenuating the inflammatory response associated with ischemia–reperfusion injury. Despite these encouraging findings, tocilizumab is not yet approved for ACS treatment and remains investigational in this context.

Ziltivekimab, a novel monoclonal antibody targeting IL-6 has shown strong anti-inflammatory effects in patients with chronic kidney disease and high cardiovascular risk by significantly lowering hsCRP and other inflammatory markers [200]. It is being developed specifically for atherosclerosis but has not yet received approval for ACS or cardiovascular indications. Several major ongoing clinical trials are investigating ziltivekimab, a fully human monoclonal antibody targeting the interleukin-6 (IL-6) ligand, for its ability to reduce inflammation and cardiovascular risk in high-risk patient populations.

The RESCUE trial was a phase 2, randomized, double-blind study evaluating the effects of ziltivekimab, a fully human monoclonal antibody targeting the IL-6 ligand, in patients at high atherosclerotic risk—specifically those with moderate to severe CKD and elevated hsCRP ≥ 2 mg/L. Ziltivekimab markedly reduced biomarkers of inflammation and thrombosis relevant to atherosclerosis in a high-risk CKD population [201].

The magnitude of hsCRP reduction (dose-dependent and marked reduction in hsCRP at 12 weeks: 7.5 mg: 77% median reduction, 15 mg: 88% median reduction, 30 mg: 92% median reduction vs. Placebo: 4% median reduction) was about twice that seen with canakinumab (an IL-1β inhibitor) in the CANTOS trial. The safety and efficacy profile supports ongoing investigation in large-scale cardiovascular outcomes trials, such as the ZEUS trial, to determine if these biomarker changes translate into fewer cardiovascular events [202].

#### Ongoing Trials

The ZEUS Trial (NCT05021835) is a large, phase-3, double-blind, placebo-controlled trial including 6200 adults with acute cardiovascular disease, chronic kidney disease and systemic inflammation (documented by elevated hs-CRP levels), to whom ziltivekimab, 15 mg or placebo is administered subcutaneously once monthly in addition to standard care. The primary aim is to determine whether ziltivekimab reduces major adverse cardiovascular events by serial follow-up for up to 4 years [202,203].

The ARTEMIS trial (NCT06118281) is a phase 3, randomized, placebo-controlled trial that investigates the effect of ziltivekimab, 15 mg or placebo, when initiated during hospital admission for acute myocardial infarction (STEMI), on the recurrence of acute cardiovascular events post-MI.

The HERMES Trial (NCT05636176) is a phase 3, randomized, placebo-controlled trial that explores the effects of ziltivekimab in improving symptoms and outcomes in heart failure [204].

Other anti-inflammatory agents like colchicine reduce IL-6 indirectly and are used off-label in cardiovascular disease. However, colchicine is not a direct IL-6 inhibitor nor approved specifically for IL-6 targeting in ACS.

In summary, while IL-6 is a validated therapeutic target in ACS due to its role in inflammation and plaque instability, no IL-6-targeted medication is currently approved for ACS treatment. Agents like tocilizumab and ziltivekimab are under clinical investigation and may become approved in the future pending results from large-scale cardiovascular outcome trials.

### 5.12. Lp-PLA2 Inhibition

Darapladib is an oral, selective inhibitor of lipoprotein-associated phospholipase A2 (Lp-PLA2), an enzyme implicated in the inflammatory processes of atherosclerosis and ACS [205]. An observational study from SOLID—TIMI 52 (Stabilization of Plaque Using Darapladib-Thrombolysis in Myocardial Infarction 52) showed that increased IL-6 was associated with increased risk of future cardiovascular events, independent of the risk factors present and baseline hsCRP level [29]. This has also been demonstrated in several meta-analyses, which show that increased levels of IL-6 and hsCRP after an acute coronary event correlate with prognosis and long-term MACE incidence, making IL-6 blockade an attractive target in the management of ACS [27].

Preclinical studies suggested that darapladib could reduce Lp-PLA2 and Rho kinase activity, lower inflammatory markers (such as CRP), improve nitric oxide production, and reduce cardiomyocyte apoptosis in animal models of atherosclerosis [205]. The most extensive and most definitive study of darapladib in ACS was the SOLID-TIMI 52 trial [206], a multinational, double-blind, placebo-controlled trial enrolling over 13,000 patients within 30 days of hospitalization for ACS. Patients received darapladib or placebo in addition to standard guideline-directed therapy and were followed for a median of 2.5 years. Darapladib did not reduce the risk of major coronary events (a composite of coronary heart disease death, myocardial infarction, or urgent coronary revascularization) compared to placebo (16.3% vs. 15.6% at 3 years; HR 1.00, *p* = 0.93). There were also no significant differences in cardiovascular death, myocardial infarction, stroke, or all-cause mortality between the darapladib and placebo groups. The safety profile was generally acceptable, though darapladib was associated with a higher incidence of odor-related concerns and diarrhea. As a result, darapladib is not approved for use in ACS, and its clinical development for this indication has been discontinued.

### 5.13. Metothrexate

Methotrexate (MTX) is a well-established anti-inflammatory and immunosuppressive drug, most commonly used in the management of autoimmune diseases such as rheumatoid arthritis (RA). Its potential cardiovascular benefits have been of increasing interest due to the recognized role of inflammation in the development and progression of atherosclerosis and ACS [207]. Multiple studies and meta-analyses have shown that methotrexate use in patients with RA is associated with a reduced risk of cardiovascular events, including ACS, compared to patients not on MTX. This cardioprotective effect is thought to be due to MTX’s ability to suppress systemic inflammation, which is a key driver of atherosclerosis and plaque instability, leading to ACS [207,208]. While the benefits of MTX in reducing cardiovascular events are apparent in patients with systemic inflammatory diseases, its role in the general population with established CAD or ACS is less specific. The Cardiovascular Inflammation Reduction Trial (CIRT) [209] investigated low-dose MTX in patients with stable CAD post-MI and did not demonstrate a significant reduction in cardiovascular events or inflammatory biomarkers compared to placebo (incidence rate, 4.13 vs. 4.31 per 100 person-years; hazard ratio, 0.96; 95% confidence interval [CI], 0.79 to 1.16). The lack of efficacy in this population may be due to lower baseline levels of inflammation compared to RA patients. In MethotrexaTE THerapy in ST-Segment Elevation MYocardial InfarctionS (TETHYS). prospective, randomized, phase 2, double-blind, placebo-controlled trial methotrexate in patients with STEMI did not reduce infarct size and further depressed LVEF at 3 months [210]. While MTX’s anti-inflammatory mechanisms (adenosine upregulation, cytokine suppression, oxidative stress reduction) are theoretically relevant to STEMI, clinical trials demonstrate no reduction in infarct size or mortality, with potential harm to ventricular function. Current evidence does not support MTX use in STEMI treatment, highlighting the complexity of targeting inflammation in acute myocardial injury [3].

### 5.14. OxLDL-Targetting

Farina et al. investigated in a phase 2a, double-blind, randomized, placebo-controlled trial the effects of orticumab, an oxLDL epitope-targetting monoclonal antibody, on coronary inflammation and residual inflammatory risk in patients with psoriasis, as oxLDL-induced macrophage activation is inhibited in atherosclerotic plaques. The study aimed to assess orticumab’s effects on both skin and coronary disease inflammation in subjects with psoriasis, as this skin condition associated with increased cardiovascular risk. Orticumab significantly reduced the Fat Attenuation Index (FAI) score in the right coronary artery (RCA) (*p* = 0.01 vs. baseline and *p* = 0.02 vs. placebo) in the subgroup of patients with elevated coronary inflammation at baseline (FAI score ≥ 50th centile) [211]. A trend towards reduction was also observed in the left circumflex (LCX) artery (*p* = 0.01 vs. baseline and *p* = 0.05 vs. placebo).

Schwab et al. report a novel use of CAR T cell technology to target atherosclerotic cardiovascular disease, specifically through murine 2D03-CAR Tregs directed against malondialdehyde-modified apolipoprotein B-100 epitope (IEI-E3 and 2D03), underscoring the importance of inducing immune tolerance to OxLDL at plaques using Tregs to stabilize or regress plaques. Targeting OxLDL with CAR Tregs at atherosclerotic lesions presents a localized, effective, and low-risk treatment alternative for refractory patients.

Compared to other therapies, OxLDL-specific CAR Treg treatment may offer long-term benefits with a single administration and lower risk of adverse reactions, enhancing its appeal for clinical use. Although this study provided positive results in murine models, this approach possesses several limitations, including differences in murine and human CAR-T cell behavior, clinical Treg therapy implementation, sorting and expanding natural Tregs and ensuring the stability of antigen-specific Tregs. However, the potential translation of 2D03-CAR Treg therapy from preclinical success to clinical application is promising, given the global functionality and success of CAR T cell manufacturing [212,213].

### 5.15. PAD4 Inhibition

Since PAD4 is a key enzyme involved in the process of NETosis, drugs targeting this enzyme hold promising potential for reducing cardiovascular risk. Experimental inhibitors, such as GSK484 and chloro-amidine, have shown positive results in murine models of myocardial infarction by reducing infarct size. This effect is attributed to the inhibition of PAD4, which decreases NETosis. By halting the NETosis process, these drugs reduce cardiac ischemia-induced apoptosis of cardiomyocytes and myocardial ischemia–reperfusion injury. Consequently, the overall cardiac function is improved [214,215].

### 5.16. GLP-1 Receptor Agonists and Inflammation

Medications that act on GLP-1 receptors are known as GLP-1 receptor agonists (GLP-1RAs), and they primarily include injectable and oral agents designed to treat type 2 diabetes and obesity by mimicking the effects of endogenous GLP-1 hormone.

These medications bind and activate GLP-1 receptors, mainly in pancreatic β cells and neurons. The result is enhanced glucose-dependent insulin secretion, inhibition of glucagon release, slowed gastric emptying, regulation of appetite, and cardioprotective effects. These actions make GLP-1RAs effective for glycemic control and weight reduction, as well as for reducing cardiovascular risk in people with diabetes and obesity [216].

GLP-1 receptor agonists have a recognized anti-inflammatory role that extends beyond their glucose-lowering and weight-management effects. These drugs modulate immune responses by acting on GLP-1 receptors expressed on several immune cells, including macrophages, monocytes, and lymphocytes [217]. GLP-1RAs suppress the production of pro-inflammatory cytokines, such as TNF-α, IL-1β, and IL-6, while increasing anti-inflammatory mediators, such as IL-10 and adiponectin. GLP-1RAs activate the AMP-activated protein kinase (AMPK) pathway, which is crucial for suppressing inflammation and promoting metabolic equilibrium [218]. These agents can also shift macrophages from a pro-inflammatory M1 state to an anti-inflammatory M2 phenotype, restoring immune balance in tissues.

GLP-1 receptor agonists, such as liraglutide, semaglutide, and exenatide, have been shown to decrease systemic inflammation by reducing circulating levels of inflammatory biomarkers, including hsCRP, TNF-α, and IL-6, in people with type 2 diabetes, obesity, and cardiovascular disease. Clinical and preclinical evidence indicate that these drugs stabilize atherosclerotic plaques by reducing monocyte adhesion and macrophage accumulation, and they slow atherosclerosis progression by modulating inflammatory pathways [217].

In a controlled trial involving men with type 2 diabetes, weekly semaglutide administration for 6 months resulted in statistically significant decreases in circulating TNF-α and IL-6, demonstrating direct anti-inflammatory benefits [219]. Evidence synthesis across multiple RCTs shows that semaglutide reduces hsCRP by 12–20%, TNF-α by 15–40%, and IL-6 by 10–25%, independent of weight loss or glycemic effects, in patients with type 2 diabetes and obesity.

The SELECT trial, involving over 17,000 high-risk cardiovascular patients without diabetes, found semaglutide reduced high-sensitivity CRP by 12–20% within 4–8 weeks of treatment, even before significant weight loss [220]. Large cardiovascular outcome trials (such as SUSTAIN-6) included patients with prior myocardial infarction or established atherosclerotic disease. They demonstrated significant reductions in major adverse cardiovascular events with semaglutide, but these studies did not specifically focus on inflammation after recent ACS [221]. Post hoc analyses and contemporary registries have shown benefits in reducing recurrent coronary events in patients with prior MI or revascularization. Still, inflammation as a primary outcome was not the focus.

GLP-1 receptor agonists are increasingly considered for secondary prevention after ACS, supported by growing evidence of cardiovascular and anti-inflammatory benefits in high-risk patients. GLP-1 receptor agonists (liraglutide, semaglutide, dulaglutide, exenatide) are recommended for patients with type 2 diabetes and established atherosclerotic cardiovascular disease, including those with prior MI or ACS, to reduce major MACE beyond glucose lowering [222].

RCTs and cohort studies show reductions in hsCRP, IL-6, TNF-α, and improvement in endothelial function, which likely contribute to stabilization of plaque and reduction in recurrent events. Evidence from observational studies and post hoc analyses in MI survivors (mostly liraglutide, some semaglutide) shows reduced risk of recurrent MI, CV mortality, reduced infarct size, and attenuation of LV remodeling when started as early as hospital discharge or soon after ACS. Trials in ACS patients, especially post-PCI STEMI, demonstrate significant myocardial salvage and reduction in inflammatory markers when GLP-1 agents are added as adjunct therapy in the acute phase (within days to weeks) [223]. Guidelines and trials suggest safe initiation during acute hospitalization or soon after discharge, optimizing patient motivation and education. For best results, start GLP-1RAs within the first days to weeks post-ACS unless contraindicated by instability, acute heart failure, GI intolerance, or renal dysfunction. Most evidence in ACS and high CV risk is for liraglutide, semaglutide, and dulaglutide, with largest CV event reduction and anti-inflammatory data for liraglutide and semaglutide [224].

Both ESC [1] and ACC/AHA guidelines [225] recommend GLP-1 receptor agonists as part of the secondary prevention strategy for patients with acute coronary syndrome who have type 2 diabetes or very high cardiovascular risk. These agents, such as semaglutide, liraglutide, and dulaglutide, are supported by evidence showing they reduce major adverse cardiovascular events, with anti-inflammatory benefits contributing to plaque stabilization and vascular protection. Initiation of therapy is appropriate after stabilization, either during hospitalization or in the early post-discharge period. Use in overweight or obese patients without diabetes is now considered reasonable, especially semaglutide, considering recent trials like SELECT that show event reduction in this group. For ACS survivors without diabetes or obesity, routine use is not guideline-recommended. Still, it may be considered in select individuals at very high risk, based on individualized evaluation and shared decision-making [226].

The mechanisms of the previously described anti-inflammatory therapies are summarized in Table 1.

### 5.17. C-Reactive Protein Apheresis in Acute Coronary Syndromes

C-reactive protein (CRP) plays a detrimental role in acute myocardial infarction (AMI) by mediating tissue damage and potentially worsening clinical outcomes. Acting as a primitive antibody, CRP binds to viable hypoxic myocardial cells, marking them for phagocytosis and exacerbating myocardial injury [227]. Plasma CRP levels rise within 48 h following myocardial infarction and inversely correlate with prognosis in acute coronary syndromes (ACS), suggesting that therapeutic CRP reduction may improve outcomes [227,228].

The CAMI-1 trial, a pilot study involving 83 STEMI patients, investigated the safety and efficacy of CRP apheresis performed at 24, 48, and 72 h post-AMI. The primary endpoint was reduction in myocardial infarction size as determined by cardiac magnetic resonance imaging (CMR). In the control group, CRP concentration directly correlated with larger infarct size (*p* = 0.002) and decreased myocardial function (*p* ≤ 0.001). Notably, patients who underwent CRP apheresis showed no correlation between CRP levels and infarct size (*p* = 0.66) or left ventricular dysfunction (*p* = 0.79), suggesting that early CRP removal may attenuate myocardial damage and preserve cardiac function [229].

Emerging evidence supports the potential benefit of CRP apheresis in non-ST-elevation ACS (NSTE-ACS) as well. In a case series of 10 patients with NSTE-ACS complicated by cardiogenic shock, 2–4 sessions of CRP apheresis were performed following culprit vessel percutaneous coronary intervention (PCI) and standard medical therapy, resulting in no 30-day mortality and only 10% (1/10) mortality at one year. Another case series evaluated 7 NSTE-ACS patients with significantly elevated plasma CRP levels (>10 mg/L) who underwent PCI followed by 2–4 cycles of CRP apheresis until CRP levels plateaued at 2–3 days post-PCI. The procedure was well tolerated with no relevant adverse effects, no in-hospital mortality, and no 30-day mortality. Notably, most patients (6/7) were male, and 4/7 had left circumflex artery stenosis or occlusion; one patient had concurrent SARS-CoV-2 infection, representing a potential confounder [229]. While these preliminary results are encouraging, long-term data on mortality and heart failure development remain unavailable. Ongoing clinical trials are currently evaluating CRP apheresis in patients undergoing coronary artery bypass grafting (CABG), which may further clarify its role in reducing inflammation-mediated myocardial injury and improving long-term cardiovascular outcomes (NCT04745468).

### 5.18. Main Gaps in Knowledge and Future Perspectives

There is no validated framework for identifying which ACS patients have “residual inflammatory risk” based on dynamic biomarkers, genetic signatures, or immune cell phenotypes. Extensive trials have not prospectively applied a multi-marker, multi-omics strategy for targeted therapy allocation. The ideal window for initiating anti-inflammatory agents—whether in the acute phase (hours to days) or chronic phase (weeks to months post-ACS)—remains unclear. Data suggest that efficacy and safety may differ based on timing, but definitive recommendations are lacking.

Significant trial limitations in the field of anti-inflammatory therapy for ACS include heterogeneity in patient selection and timing of intervention, unclear biomarkers to guide therapy, limited mechanistic endpoints, and safety concerns. These limitations impact the strength and applicability of clinical evidence for inflammation-targeted strategies.

Most trials focus on broad clinical endpoints (MACE, MI, death), with limited mechanistic endpoints such as plaque imaging, immune cell dynamics, or signaling pathway modulation. The specific molecular mechanisms underlying the benefits or harms of anti-inflammatory therapy remain underexplored.

Many trials enroll broad ACS populations without stratifying by residual inflammatory risk (e.g., baseline hsCRP, IL-6). This can dilute the apparent efficacy of treatments, as only some subgroups benefit (such as those with persistently elevated inflammation after ACS).

Randomization and treatment initiation times vary widely. Colchicine trials, for example, range from administration within hours post-infarction (CLEAR SYNERGY) to weeks later (COLCOT), resulting in inconsistent outcomes and challenging direct comparisons. Early therapy may interfere with beneficial inflammation needed for healing, while late therapy may miss the optimal window for efficacy.

Concomitant treatments (statins, antiplatelets, aggressive lipid-lowering) vary and may independently reduce inflammation, masking or amplifying the effects of the study drug. Differences in population characteristics and event rates may lead to variability across studies. Increased risk of infections (canakinumab) and unexplained non-cardiovascular mortality (colchicine, COPS trial) highlight the need for careful long-term safety assessment. Some adverse effects only become apparent with larger or longer studies.

Despite promising results, guideline recommendations for anti-inflammatory therapy remain cautious; robust, biomarker-driven criteria for patient selection are lacking, and current evidence does not support routine use in all ACS patients.

These challenges highlight why inflammation-targeted therapy is not yet standard for all ACS patients and indicate areas where future trial design can be improved.

Experimental and emerging molecular targets (NETosis, PAD4, pro-resolving mediators, epigenetic regulators, and immune cell engineering) have not yet been translated into adequately powered human trials. Their clinical potential and patient selection criteria require systematic evaluation. While genetic predisposition and polygenic risk scores hold promise, their integration into routine ACS management and clinical trial design is preliminary, and there is no consensus on threshold or clinical utility.

Most large trials are limited in duration and do not provide full assessment of long-term safety, especially concerning immunosuppression, infection risk, and non-cardiovascular mortality.

Current guidelines do not universally endorse anti-inflammatory therapy in ACS outside secondary prevention with statins; recommendations await stronger evidence from well-designed, biomarker- and genetics-guided trials.

Addressing these gaps requires innovative integrative research—using multi-omics, immune profiling, adaptive trial designs, and mechanistic endpoints—to move from broad anti-inflammatory strategies to precision inflammation management in ACS patients.

A unique perspective in the field of ACS inflammation would emphasize the shift toward precision anti-inflammatory therapy, focusing on individualized risk stratification and targeting distinct inflammatory pathways based on patient-specific biomarkers and timing, rather than one-size-fits-all approaches. Original hypotheses could be centered on new molecular mechanisms, cellular interactions, or therapeutic windows that remain underexplored or controversial.

Recent advances in ACS research support the concept that anti-inflammatory therapies should not be applied indiscriminately, but instead rationed to patients with demonstrable residual inflammatory risk after event recovery. Multiplex panels measuring hsCRP, IL-6, the neutrophil-to-lymphocyte ratio, and novel genetic or epigenetic markers should guide the initiation and choice of immunomodulation. Furthermore, the therapeutic window for suppressing pathologic inflammation while preserving reparative processes is likely narrow and varies among individuals. Ongoing studies of NLRP3 inhibitors, NETosis blockers, adaptive immune modulators (e.g., low-dose IL-2, GLP-1RA), and advanced profiling will clarify how targeted therapy may optimize outcomes. Ultimately, integrated approaches combining anti-inflammatory, lipid-lowering, and metabolic interventions—each tailored to patient-specific molecular and immune profiles—may redefine secondary prevention in ACS.

Several promising but underexplored molecular targets for anti-inflammatory therapy in acute coronary syndromes (ACS) include NETosis and PAD4-mediated mechanisms, modulation of immune cell subsets, epigenetic regulators, and advanced chemokine signaling networks. These targets offer new strategies beyond traditional cytokine blockade and could help refine patient selection and optimize efficacy.

Neutrophil extracellular traps (NETs) directly contribute to plaque rupture, thrombosis, and microvascular injury during ACS. PAD4 (peptidyl-arginine-deiminase-4) is essential for NET formation, and its pharmacologic inhibition (e.g., GSK484, chloro-amidine) in preclinical models reduces infarct size, cardiac dysfunction, and prothrombotic risk. Targeting NETosis in ACS clinical trials is ongoing, but human data are limited.

Specialized pro-resolving mediators (SPMs)—such as lipoxins, resolvins, and protectins—actively terminate inflammation and promote tissue repair. Their therapeutic modulation represents a unique, underutilized approach to control inflammation and guide healing after myocardial infarction.

Fine-tuning specific immune cell subsets (e.g., promoting regulatory T cells with low-dose IL-2 or inhibiting CD8+ granzyme B activity) offers highly targeted options in ACS immune response. Agents such as CAR-Tregs targeting oxidized LDL show preclinical promise for local vascular tolerance induction.

Modulating microRNA profiles (e.g., miR-33, miR-146a), SUMOylation, phosphorylation, and ubiquitin-mediated signaling in inflammatory cells may regulate NLRP3 activation or downstream effects, potentially providing patient-specific intervention windows.

Drugs targeting less-studied chemokines (such as CCL2, CXCL1, CXCL5) and their receptors (ACKR1, CCR2, CXCR2) could selectively inhibit pathogenic leukocyte recruitment, extracellular matrix remodeling, and fibrosis post-ACS. There is preliminary evidence for protective and pathogenic roles depending on context and timing.

Monoclonal antibodies such as orticumab, or engineered regulatory immune cells (CAR-Tregs), targeting oxidized LDL and its epitopes could suppress local coronary artery inflammation and stabilize vulnerable plaques, but human application remains experimental.

Beyond established IL-1 antagonists, selective targeting of NLRP3 with agents like MCC950 and OLT1177 could offer more nuanced inflammatory control during ACS, especially in individuals with specific inflammasome activation profiles.

GLP-1RAs modulate immune responses and metabolic-inflammatory crosstalk and may provide protection, especially when timing is optimized during ACS hospitalization. Their effects on immune cell polarization (macrophage M2 vs. M1 shift) and vascular inflammation warrant further exploration.

These molecular pathways are beginning to draw research attention and represent highly innovative strategies for precision inflammation targeting in ACS. Including discussion or hypotheses around these targets can substantially increase the originality and impact of a review or research proposal in this field.

Emerging genetic profiling and polygenic risk models have a growing role in refining risk stratification and guiding therapeutic decisions for inflammation-targeted interventions in acute coronary syndromes (ACS). They allow for the identification of individual genetic variants and cumulative risk scores that influence disease susceptibility, residual inflammatory risk, and variable treatment response.

Advanced genetic profiling can identify patients with inherited susceptibility to ACS or more aggressive atheroinflammatory phenotypes, using genetic markers (e.g., variants in IL-6R, NLRP3, TLR4, CRP, and other inflammation-related genes). Polygenic risk scores (PRS), integrating hundreds or thousands of individual genetic variants, allow for robust prediction of overall ASCVD risk and can reveal those at increased residual inflammatory risk despite optimal conventional therapy.

Genetic variation affects both disease mechanisms and response to therapy. For example, patients carrying specific genetic variants or expression profiles in inflammasome or cytokine pathways may derive greater benefit from targeted anti-inflammatory drugs or may require dose adjustment or alternative strategies. This can help to optimize individualized care and avoid unnecessary treatment in those unlikely to benefit.

Genetic profiling supports the identification of novel mechanistic targets and enables more precise selection of trial populations. For example, enriched recruitment of patients with high polygenic inflammation risk or specific gene expression signatures (e.g., high NLRP3 activation) can improve trial yield and clarity of results. In addition, integrating genetic profiling with multiplex inflammatory biomarker panels supports a “multi-omics” approach for patient selection and risk prediction.

Differences among ethnic groups, family history, and unexplained outcomes may be clarified through genetic analysis, improving understanding of why some patient groups respond dramatically while others do not.

Routine genetic and polygenic profiling may soon complement standard clinicopathologic risk scores, further sharpening the selection of patients for anti-inflammatory therapies, whether colchicine, IL-1 antagonists, or newer agents.

Combining genetic predisposition (from PRS) with dynamic inflammatory markers (hsCRP, IL-6, NLRP3, etc.) can identify “super-responders” and help personalize both timing and intensity of inflammatory modulation post-ACS. This approach marks a key transition in ACS management from one-size-fits-all secondary prevention to precision medicine, maximizing therapeutic benefit while minimizing risk and healthcare costs.

Inflammation is now recognized as a fundamental driver of acute coronary syndromes (ACS), influencing plaque instability, thrombosis, and post-infarction myocardial injury. Recent discoveries offer critical insights into pathophysiology, highlight the prognostic value of inflammatory biomarkers, and identify new therapeutic targets, moving the field beyond traditional risk factor management and antithrombotic therapies.

Inflammatory mechanisms in ACS involve complex interactions among innate and adaptive immune cells, including neutrophil extracellular trap formation, macrophage polarization, T helper subset imbalances, and overactivation of inflammatory cytokines and chemokines. Multiple biomarkers—such as C-reactive protein, interleukin-6, and the neutrophil-to-lymphocyte ratio—demonstrate value for prognostic risk stratification. Modern anti-inflammatory therapies, including colchicine and IL-1 inhibitors, have shown clinical efficacy in reducing recurrent events, though outcomes can depend on timing, dosing, and patient selection. Novel agents that target the NLRP3 inflammasome, NETosis, Th17/Treg imbalance, and IL-6 are under investigation, promising greater precision in managing residual inflammatory risk beyond lipid-lowering [230,231]. New anti-inflammatory medications such as colchicine, canakinumab, anakinra, and ziltivekimab show promise for reducing residual inflammation and cardiovascular events after ACS, with potential overlap in benefits for myocarditis due to shared inflammatory pathways. However, the 2025 guidelines (ESC for myocarditis/pericarditis and ACS) do not endorse their routine use for myocarditis post-ACS, limiting colchicine to Class IIb for selected ACS cases with uncontrolled risk and excluding others pending trial data from CLEAR [232], TACTIC [233], and ARTEMIS (NCT06118281). 2025 ESC myocarditis guidelines prioritize EMB/CMR for etiology before immunosuppression, omitting these agents except NSAIDs/colchicine for pericarditis features [234]. Theoretical benefit via IL-6 pathway inhibition exists for inflammatory myocarditis subtypes, but the absence of safety/efficacy data in EMB-proven cases contraindicates off-label use per the 2025 ESC guidelines. Risks include infections and HF exacerbation, so dedicated trials are desirable. No large-scale clinical trials specifically test IL-6 inhibitors such as tocilizumab, sarilumab, or ziltivekimab directly for the treatment of primary myocarditis. Evidence is limited to small studies, case reports, and trials in related conditions, such as COVID-19 myocarditis, immune checkpoint inhibitor (ICI)-associated myocarditis, or post-MI inflammation, which show potential benefits in reducing CRP and improving ejection fraction but lack robust outcome data.

An ideal anti-inflammatory medication post-ACS targets residual inflammation (e.g., hsCRP > 2 mg/L) via specific pathways, such as IL-6 or NLRP3, to reduce MACE by ≥25% while maintaining hemostatic neutrality for DAPT compatibility. It achieves rapid onset (<24 h CRP reduction), oral/short IV dosing, long half-life, and safety in CKD/HF subsets without elevating infection or GI risks.

The ≥25% MACE reduction threshold for ideal post-ACS anti-inflammatories sets a clinically meaningful benchmark, surpassing current agents like colchicine (7–23% relative risk reduction, often heterogeneous without mortality benefit). This aligns with landmark trials (e.g., CANTOS: canakinumab 20–25% in hsCRP-high; statins 25–30%) and regulatory standards for guideline endorsement (Class I/IIa), ensuring that the net benefit outweighs risks such as GI upset or infection.

Future anti-inflammatory treatment after ACS will shift toward personalized, pathway-specific therapies targeting IL-6, IL-1β, and the NLRP3 inflammasome, integrated with biomarkers such as hsCRP/IL-6 for patient selection. Expect multi-targeted regimens combining these with statins, SGLT2i, and antithrombotics, emphasizing rapid onset, myocardial salvage, and a ≥25% reduction in MACE in high-inflammation subsets. New biomarkers and AI will play pivotal roles in future anti-inflammatory treatment after ACS by enabling precise patient selection, timing, and monitoring of therapies like IL-6 inhibitors. Emerging markers such as ARG1, HECW2, PFKFB3, and systemic indices (SIRI, NLR) outperform hsCRP alone in predicting MACE and immune infiltration, guiding personalized care beyond standard inflammation thresholds. AI models (e.g., machine learning via WGCNA/SVM-RFE [Weighted Gene Co-expression Network Analysis/Support Vector Machine—Recursive Feature Elimination], deep learning for miRNA-mRNA networks) identify biomarker panels from GEO data to predict inflammation trajectories and therapy response. AI models integrate multi-omic (genomics, proteomics, metabolomics) and clinical data to predict ACS risk through dimensionality reduction, feature fusion, and supervised learning frameworks such as MLPs, transformers, or ensemble methods. This enables precise stratification of inflammation-driven MACE risk, outperforming traditional scores. AI excels at identifying ACS patient subgroups benefiting most from anti-inflammatory therapy by clustering multi-omic, clinical, and imaging data into endotypes with differential treatment responses. Models like ANNs or transformers outperform GRACE/TIMI (C-index 0.73–0.85 vs. 0.67), reclassifying 6–18% more patients via NRI for high-inflammation phenotypes (e.g., hsCRP > 2 mg/L, elevated IL-6). Prospective trials will validate AI-stratified protocols, enabling guideline shifts and reducing overtreatment risks, such as infection.

### 5.19. Clinical Implications and Current Limitations

The implementation of anti-inflammatory strategies in ACS is rapidly evolving, with trials like COLCOT and CANTOS validating the reduction in cardiovascular events through selective targeting of inflammation. However, broad application in clinical practice remains limited by variable efficacy in different patient populations, uncertain optimal timing and duration, lack of validated biomarkers for patient selection, and concerns about long-term safety—especially infection risk. Recent neutral or safety-concerning findings in large trials (e.g., CLEAR SYNERGY for colchicine) underscore the need to tailor therapy to individual inflammatory profiles and to carefully monitor for adverse effects. While current ESC and ACC/AHA guidelines acknowledge the role of inflammation in ACS, they do not yet recommend routine use of anti-inflammatory agents outside of statin therapy, except in select high-risk cases.

Looking ahead, the future of ACS care rests on integrating precision anti-inflammatory interventions alongside optimized lipid management and secondary prevention. Multiplex biomarker panels, genetic risk scores, and advanced immune profiling are essential for identifying candidates with residual inflammatory risk who are most likely to benefit from targeted therapies. Ongoing large-scale trials and real-world cohort studies will clarify optimal timing, duration, and safety. Moving forward, research should focus on leveraging systems biology, multi-omics, and adaptive trial design to refine therapeutic selection, minimize harm, and further personalize cardiovascular care.

In conclusion, inflammation represents both a major residual risk and an exciting therapeutic frontier in ACS. The integration of individualized anti-inflammatory strategies with existing guideline therapies has the potential to profoundly improve outcomes for patients worldwide, provided future studies continue to address gaps in evidence, refine patient selection, and ensure long-term safety.

## Figures and Tables

**Table 1:** Currently studied anti-inflammatory therapies in acute coronary syndromes.

Drug	Effects	Observations
Aspirin	Inhibits COX-1 and COX-2 enzymes Reduces inflammatory cytokines (TNF-α, IL-6, IL-1β) and MCP-1 Acetylates COX-2 to produce aspirin-triggered lipoxin A4 (potent anti-inflammatory mediator) Stimulates eNOS and reduces oxidative stress in vascular cells Inhibits platelet activation and reduces platelet-leukocyte aggregation Decreases endothelial activation and expression of adhesion molecules Protects LDL from oxidative modification Inhibits free radical formation and lipid peroxidation	Antiplatelet effects achieved at low doses (75–100 mg/day); anti-inflammatory effects at higher doses (300 mg/day)
Statins	Decrease inflammatory markers: IL-1β, CRP, IL-6, and TNF-α Inhibit leukocyte recruitment and adhesion to endothelium by reducing adhesion molecule expression (ICAM-1, VCAM-1, E-selectin, P-selectin) Decrease MPO activity and oxidative stress Reduce neutrophil activation and infiltration into atherosclerotic plaques Stabilize atherosclerotic plaques by reducing inflammation	Rosuvastatin (20 mg): 44% CRP reduction Atorvastatin (20 mg): 30% CRP reduction Benefits individuals without hyperlipidemia but with elevated inflammation (hsCRP ≥ 2 mg/L)
PCSK9 Inhibitors (evolocumab, alirocumab)	Reduce LDL cholesterol and inflammatory markers Decrease oxidative stress Stabilize atherosclerotic plaques Complement statin therapy for lipid and inflammation control	
Colchicine	Inhibits microtubule polymerization in neutrophils and monocytes Reduces neutrophil chemotaxis and adhesion to endothelium Decreases NLRP3 inflammasome activation Reduces IL-1β and IL-18 production Lowers inflammatory markers (hsCRP reduced from 4.27 mg/dL to 2.98 mg/dL) Reduces risk of major adverse cardiovascular events (MACE)	Reduces IL-1β and IL-18 production Lowers inflammatory markers (hsCRP reduced from 4.27 mg/dL to 2.98 mg/dL) Reduces risk of major adverse cardiovascular events (MACE)
Canakinumab (IL-1β inhibitor)	Monoclonal antibody targeting IL-1β Reduces first and total major cardiovascular events Significantly decreases hsCRP levels (median reduction from 4.2 mg/L) Reduces recurrent cardiovascular events in patients with residual inflammatory risk (hsCRP ≥ 2 mg/L) Inhibits IL-1β pathway, preventing downstream inflammatory cascade Reduces risk of recurrent MI, stroke, and cardiovascular death	Dose-dependent effect (50 mg, 150 mg, 300 mg subcutaneous every 3 months) 150 mg dose showed optimal risk–benefit profile Side effects: increased risk of fatal infections and sepsis
Anakinra (IL-1 receptor antagonist)	Recombinant IL-1 receptor antagonist blocking both IL-1α and IL-1β effects Reduces systemic inflammation in STEMI patients Decreases incidence of new-onset heart failure (9.4% vs. 25.7% placebo, *p* = 0.03) May reduce complications such as heart failure post-STEMI	No significant difference in infection rates compared to placebo (14% vs. 14%) No significant impact on end-systolic volume or ejection fraction in some studies Effectively reduces inflammation but cardiovascular benefit requires further validation
Low-dose IL-2	Expands regulatory T cells (Tregs) Restores Th17/Treg balance Has immune-suppressive and anti-inflammatory properties Promotes immune homeostasis and plaque stabilization	Under investigation for cardiovascular applications
Tocilizumab (IL-6 receptor inhibitor)	Monoclonal antibody against IL-6 receptor Significantly reduces inflammatory biomarkers (hsCRP, IL-6) Reduces troponin T area under the curve (AUC) in AMI patients Less microvascular obstruction observed Well tolerated in acute settings May reduce myocardial damage in STEMI patients undergoing PCI	Single dose (280 mg IV) before PCI reduces myocardial injury Not yet approved for ACS treatment; remains investigational
Ziltivekimab (IL-6 ligand inhibitor)	Novel monoclonal antibody targeting IL-6 ligand Strong anti-inflammatory effects in chronic kidney disease and atherosclerosis Reduces hsCRP levels by approximately 80–90% CRP reduction about twice that seen with canakinumab	Monthly subcutaneous injections May offer superior inflammation control compared to IL-1 inhibitors
Methotrexate (MTX)	Anti-inflammatory and immunosuppressive drug Reduces leukocyte adhesion Inhibits pro-inflammatory cytokine production Reduces CD4+, CD8+ T cell and NK cell activation Associated with reduced cardiovascular events in rheumatoid arthritis patients	Weekly dosing (15–20 mg) used in cardiovascular studies In STEMI patients: did not reduce IL-1β, IL-6, or hsCRP at 24 h or 30 days TETHYS trial showed no reduction in MACE in STEMI patients
NLRP3 Inflammasome Inhibitors (OLT1177, MCC950)	Prevent IL-1β and IL-18 release from foam cells and macrophages Reduce pyroptosis and inflammatory cell death Limit plaque inflammation and instability	Under investigation for cardiovascular applications
PAD4 Inhibitors	Target Protein Arginine Deiminase 4 (PAD4) Inhibit neutrophil extracellular trap (NET) formation Reduce thrombogenic NETs in ruptured plaques Decrease NET-mediated platelet activation and thrombosis	Experimental agents under development
Orticumab (OxLDL-targeting antibody)	Monoclonal antibody targeting oxidized LDL (oxLDL) epitopes Inhibits oxLDL-induced macrophage activation in atherosclerotic plaques Significantly reduces Fat Attenuation Index (FAI) score in right coronary artery (RCA) (*p* = 0.01 vs. baseline, *p* = 0.02 vs. placebo) Trend towards reduction in left circumflex (LCX) artery (*p* = 0.01 vs. baseline, *p* = 0.05 vs. placebo) Reduces coronary inflammation and residual inflammatory risk Targets oxidized LDL to reduce inflammatory oxLDL-mediated injury Addresses both skin and coronary disease inflammation	Effective in patients with elevated coronary inflammation at baseline (FAI score ≥ 50th centile) Tested in phase 2a trials in patients with psoriasis (associated with increased cardiovascular risk)
GLP-1 Receptor Agonists (liraglutide, semaglutide, dulaglutide)	Suppress pro-inflammatory cytokines (TNF-α, IL-1β, IL-6) Increase anti-inflammatory mediators (IL-10, adiponectin) Activate AMP-activated protein kinase (AMPK) pathway, inhibiting NF-κB Shift macrophages from pro-inflammatory M1 to anti-inflammatory M2 phenotype Reduce oxidative stress and endothelial dysfunction Reduce hsCRP by 15–40% and IL-6 by 10–25% (independent of weight loss) Cardiovascular benefits in high-risk patients with type 2 diabetes Reduce MACE, cardiovascular death, and heart failure hospitalization	ESC and ACC/AHA guidelines recommend for secondary prevention in ACS with diabetes Best results when started within first days to weeks after ACS Injectable and oral formulations available

## Data Availability

No new data were created or analyzed in this study. Data sharing is not applicable to this article.

## Abbreviations

The following abbreviations are used in this manuscript:

ACS	Acute Coronary Syndrome
AKT	Protein Kinase B
AMI	Acute Myocardial Infarction
ASC	Apoptosis-associated Speck-like protein containing a CARD
ATP	Adenosine Triphosphate
CAD	Coronary Artery Disease
CANTOS	Canakinumab Anti-inflammatory Thrombosis Outcomes Study
CARD	Caspase Activation and Recruitment Domain
CCL4	C-C Motif Chemokine Ligand 4
CCR5	C-C Chemokine Receptor Type 5
CD40L	CD40 Ligand
CIRT	Cardiovascular Inflammation Reduction Trial
CKD	Chronic Kidney Disease
COLCOT	Colchicine Cardiovascular Outcomes Trial
CONSORT	Consolidated Standards of Reporting Trials
COVID	Coronavirus Disease
COX	Cyclooxygenase
CRP	C-Reactive Protein
CSF	Colony-Stimulating Factor
CT	Computed Tomography
CTLA	Cytotoxic T-Lymphocyte Associated Protein
DAMP	Damage-Associated Molecular Pattern
DNA	Deoxyribonucleic Acid
ECM	Extracellular Matrix
EMT	Epithelial–Mesenchymal Transition
ERK	Extracellular Signal-Regulated Kinase
ESC	European Society of Cardiology
FAI	Fat Attenuation Index
FDA	Food and Drug Administration
FDG	Fluorodeoxyglucose
HO	Heme Oxygenase
ICAM	Intercellular Adhesion Molecule
IFN	Interferon
IL	Interleukin
IVUS	Intravascular Ultrasound
LCK	Lymphocyte-Specific Protein Tyrosine Kinase
LCX	Left Circumflex Artery
LD	Linear Dichroism
LDL	Low-Density Lipoprotein
LOX	Lipoxygenase
LRR	Leucine-Rich Repeat
LVEF	Left Ventricular Ejection Fraction
MACCE	Major Adverse Cardiac and Cerebrovascular Events
MACE	Major Adverse Cardiovascular Events
MAPK	Mitogen-Activated Protein Kinase
MCP	Monocyte Chemoattractant Protein
MHC	Major Histocompatibility Complex
MI	Myocardial Infarction
MLR	Monocyte-to-Lymphocyte Ratio
MMP	Matrix Metalloproteinase
MPO	Myeloperoxidase
MTX	Methotrexate
NACHT	Neuronal Apoptosis Inhibitory Protein (NAIP), MHC Class II Transcription Activator (CIITA), Incompatibility Locus Protein from Podospora anserina (HET-E), and Telomerase-Associated Protein (TP1)
NADPH	Nicotinamide Adenine Dinucleotide Phosphate
NET	Neutrophil Extracellular Trap
NK	Natural Killer
NLR	Neutrophil-to-Lymphocyte Ratio
NLRP3	NOD-like Receptor Protein 3
NOD	Nucleotide-binding Oligomerization Domain
NOX2	NADPH Oxidase 2
NSTEMI	Non-ST-Elevation Myocardial Infarction
PAD4	Peptidylarginine Deiminase 4
PAMP	Pathogen-Associated Molecular Pattern
PCI	Percutaneous Coronary Intervention
PD	Programmed Cell Death
PET	Positron Emission Tomography
PI3K	Phosphoinositide 3-Kinase
PLR	Platelet-to-Lymphocyte Ratio
PMN	Polymorphonuclear Neutrophil
PPAR	Peroxisome Proliferator-Activated Receptor
RA	Rheumatoid Arthritis
RCA	Right Coronary Artery
RCT	Randomized Controlled Trial
RDW	Red Cell Distribution Width
RIR	Residual Inflammatory Risk
RNA	Ribonucleic Acid
ROS	Reactive Oxygen Species
RPR	Red Cell Distribution Width-to-Platelet Ratio
SII	Systemic Immune-Inflammation Index
SIRI	Systemic Inflammation Response Index
STEMI	ST-Elevation Myocardial Infarction
TCR	T-Cell Receptor
TGF	Transforming Growth Factor
TIMI	Thrombolysis in Myocardial Infarction
TLR	Toll-Like Receptor
TNF(R)	Tumor Necrosis Factor (Receptor)
Treg	Regulatory T Cell
UA	Unstable Angina
VCAM	Vascular Cell Adhesion Molecule
VEGF	Vascular Endothelial Growth Factor
WBC	White Blood Cell
